# Inhibition of Malaria Infection in Transgenic Anopheline Mosquitoes Lacking Salivary Gland Cells

**DOI:** 10.1371/journal.ppat.1005872

**Published:** 2016-09-06

**Authors:** Daisuke S. Yamamoto, Megumi Sumitani, Katsumi Kasashima, Hideki Sezutsu, Hiroyuki Matsuoka

**Affiliations:** 1 Division of Medical Zoology, Department of Infection and Immunity, Jichi Medical University, Yakushiji, Shimotsuke, Tochigi, Japan; 2 Transgenic Silkworm Research Unit, Institute of Agrobiological Sciences, National Agriculture and Food Research Organization, Owashi, Tsukuba, Ibaraki, Japan; 3 Division of Functional Biochemistry, Department of Biochemistry, Jichi Medical University, Yakushiji, Shimotsuke, Tochigi, Japan; University of Nottingham, UNITED KINGDOM

## Abstract

Malaria is an important global public health challenge, and is transmitted by anopheline mosquitoes during blood feeding. Mosquito vector control is one of the most effective methods to control malaria, and population replacement with genetically engineered mosquitoes to block its transmission is expected to become a new vector control strategy. The salivary glands are an effective target tissue for the expression of molecules that kill or inactivate malaria parasites. Moreover, salivary gland cells express a large number of molecules that facilitate blood feeding and parasite transmission to hosts. In the present study, we adapted a functional deficiency system in specific tissues by inducing cell death using the mouse Bcl-2-associated X protein (Bax) to the Asian malaria vector mosquito, *Anopheles stephensi*. We applied this technique to salivary gland cells, and produced a transgenic strain containing extremely low amounts of saliva. Although probing times for feeding on mice were longer in transgenic mosquitoes than in wild-type mosquitoes, transgenic mosquitoes still successfully ingested blood. Transgenic mosquitoes also exhibited a significant reduction in oocyst formation in the midgut in a rodent malaria model. These results indicate that mosquito saliva plays an important role in malaria infection in the midgut of anopheline mosquitoes. The dysfunction in the salivary glands enabled the inhibition of malaria transmission from hosts to mosquito midguts. Therefore, salivary components have potential in the development of new drugs or genetically engineered mosquitoes for malaria control.

## Author Summary

Malaria, transmitted by anopheline mosquitoes, represents an important global public health challenge. The salivary glands of mosquitoes are an attractive target tissue for malaria and vector control. We produced a transgenic strain inducing cell death in the salivary glands with a cell death effector molecule in the Asian malaria vector mosquito, *Anopheles stephensi*. This transgenic strain contained extremely low amounts of saliva. An analysis of this strain revealed that saliva plays an important role in probing as well as malaria infection in the midgut in a rodent malaria model. The dysfunction in the salivary glands enabled the inhibition of malaria transmission to mosquito midguts. Therefore, salivary components are also important in malaria control.

## Introduction

Malaria represents an important global public health challenge, and is transmitted by anopheline mosquitoes during blood feeding. Mosquito vector control is one of the most effective ways to control malaria in endemic areas. Insecticide resistance has recently been reported in mosquitoes in endemic areas, and, thus, new strategies to control mosquitoes are expected [1]. Population replacement with genetically engineered mosquitoes to block malaria transmission is anticipated to become a new vector control strategy [2,3].

When mosquitoes ingest the gametocytes of the malaria parasite, *Plasmodium* with blood, they enter into the midgut and differentiate into gametes. After fertilization, parasites transform into ookinetes. They traverse the peritrophic matrix and midgut epithelium, and differentiate into oocysts on the basal lamina. Oocysts then rupture, and the sporozoites released in the hemolymph invade the salivary glands only. They remain in this tissue until the next opportunity for blood feeding [4,5]. Therefore, the salivary gland is an effective target tissue for the expression of molecules that kill or inactivate malaria parasites using transgenic technologies. We previously reported that the transmission of malaria to hosts was markedly reduced in transgenic mosquitoes expressing a single-chain antibody (scFv) to the malaria circumsporozoite protein [6].

On the other hand, the salivary glands of adult female mosquitoes play an important role in blood feeding and the transmission of the malaria parasite to humans. Mosquito saliva is considered to contain a large number of different molecules that facilitate blood feeding [7,8]. Furthermore, the cells of salivary glands have been suggested to contain molecules that interact with sporozoites because malaria sporozoites have been shown to specifically invade the salivary glands of anopheline mosquitoes [9–12]. Therefore, a functional analysis of the salivary glands is important for the development of genetically engineered mosquitoes to block the transmission of malaria and drugs to control blood feeding. However, the *in vivo* functions of salivary components remain largely unknown. The induction of functional deficiencies in the salivary glands is an effective approach for investigating these functions. Although the cells of the salivary glands were recently shown to enable transgene expression, the lack of a suitable effector gene has led to difficulties elucidating the functions of the salivary glands [6,13–17].

In the present study, we established a functional deficiency system in the salivary glands by inducing cell death in the Asian malaria vector, *Anopheles stephensi*. We adapted a tissue-specific mouse Bcl-2-associated X protein (Bax)-mediated cell death system [18]. Bax is a member of the Bcl-2 family in mammals [19–21]. It assembles into ring structures in the mitochondrial outer membrane (MOM). Rings of Bax perforate the membrane, and mediate MOM permeabilization, which is an essential event in cell death [22,23]. A previous study demonstrated that mouse Bax (mBax) induced stage- and tissue-specific cell death in *Drosophila melanogaster* and *Bombyx mori*, suggesting that this mBax-mediated cell death induction system is functional and a useful tool in many other insect species [18,24,25].

We produced transgenic *An*. *stephensi* overexpressing mBax under the control of the female-specific salivary gland promoter of the *anopheline antiplatelet protein* (*aapp*) gene [13]. The females of the transgenic line had abnormal salivary glands in which cells in the distal lateral lobes were prominently lysed. We examined the blood feeding behavior of this transgenic mosquito. We also investigated the susceptibility of this transgenic mosquito to parasitic infections in a rodent malaria model. We demonstrated that the functional inhibition of the salivary glands reduced the transmission of malaria to the midgut. Therefore, identifying salivary components that interact with malaria parasites for their infection in the midgut may be promising in the development of new drugs to control mosquitoes and malaria parasites. Additionally, this mBax-mediated functional deficiency system has the potential to become a powerful tool for analyzing parasite-vector interactions and developing vector control strategies for *Anopheles* mosquitoes.

## Results

### Establishment of transgenic mosquitoes expressing mBax in the salivary gland

**Fig 1:**
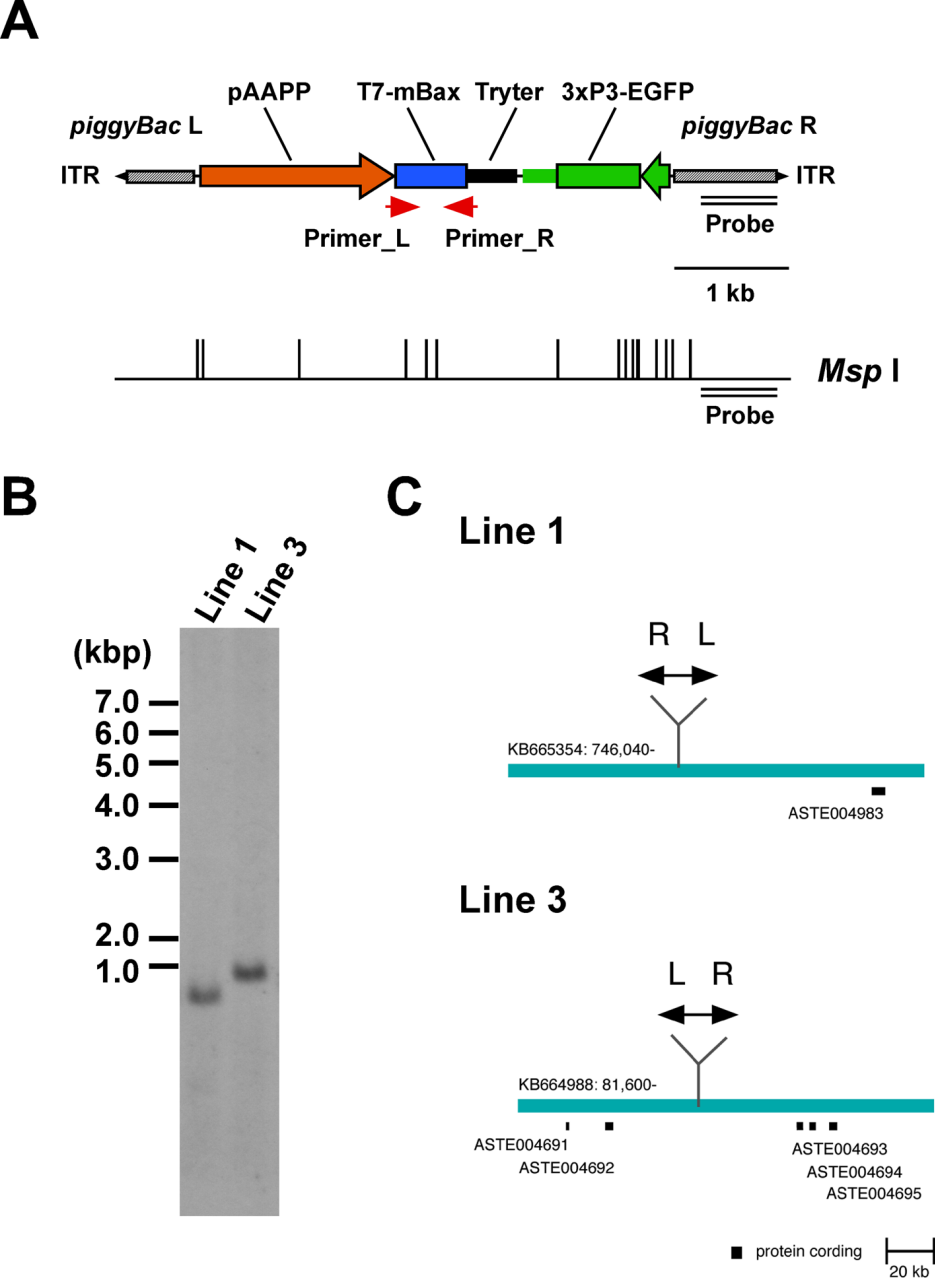
The gene structure of the *piggyBac* transformation vector, pBac[pAAPP-mBax; 3xP3-EGFP], TG mosquito lines, and insertion sites. (A) The gene construct derived from the *piggyBac*-based vector contains *piggyBac* Left-arm (L) and Right-arm (R) with an inverted terminal repeat (ITR). The *T7-mBax* gene is expressed under the control of the *An*. *stephensi aapp* promoter (pAAPP) and *An*. *gambiae trypsin* terminator (Tryter). The transformation marker, *EGFP* is expressed under the control of the *3xP3* promoter. A double line represents the probe region for a Southern blot analysis. The restriction enzyme (*Msp* I) site is represented below the scheme. The red arrow represents the primer sites for a RT-PCR analysis in Fig 2. (B) A Southern blot analysis of AAPP-mBax lines. Genomic DNA from AAPP-mBax mosquito lines (lines 1 and 3) was digested with *Msp* I, and hybridized with a fragment corresponding to the *piggyBac* R region. (C) Insertion sites of the transgene in AAPP-mBax lines 1 and 3. The blue bars show the local DNA region within each genomic scaffold. Black boxes represent the annotated protein-coding region in the VectorBase (https://www.vectorbase.org/). Double-headed arrows show the *piggyBac* construct. L: *piggyBac* Left-arm, R: *piggyBac* Right-arm.

mBax functions heterologously as a cell death effector in *D*. *melanogaster* and *B*. *mori* [18,24,25]. Therefore, a *piggyBac*-based transformation vector containing an *mBax* gene with a T7 tag under the control of the *aapp* gene promoter [13] and *An*. *gambiae trypsin* gene terminator sequences was generated (Fig 1A). The transformation vector was injected with a *piggyBac* helper vector into *An*. *stephensi* embryos. Two transgenic AAPP-mBax lines (lines 1 and 3) were established. A single copy insertion of the transgene in these lines was confirmed by a Southern blot analysis (Fig 1B). We performed inverse PCR in order to identify the insertion site, and elucidated the nucleotide sequence flanking the left arm of the *piggyBac* transgene. The insertion sites of the two transgenic lines were integrated into intergenic regions, which were separated by at least 30 kbp from the surrounding genes (Fig 1C). The two transgenic AAPP-mBax lines have been stably maintained by blood meals on mice for more than 10 generations, suggesting that the insertion of the transgene did not interfere with essential genes.

In order to examine the viability of the AAPP-mBax line, the survival rates of AAPP-mBax and wild-type adult mosquitoes after eclosion were investigated. No significant differences were observed between transgenic and wild-type mosquitoes under sugar feeding and blood feeding conditions (S1 Fig). Furthermore, the number of eggs laid and the hatchability were not significantly different between transgenic and wild-type mosquitoes (S1 Table). These results indicate that the transgene did not affect endogenous genes and the viability of mosquitoes.

### Effects of mBax on salivary glands

**Fig 2:**
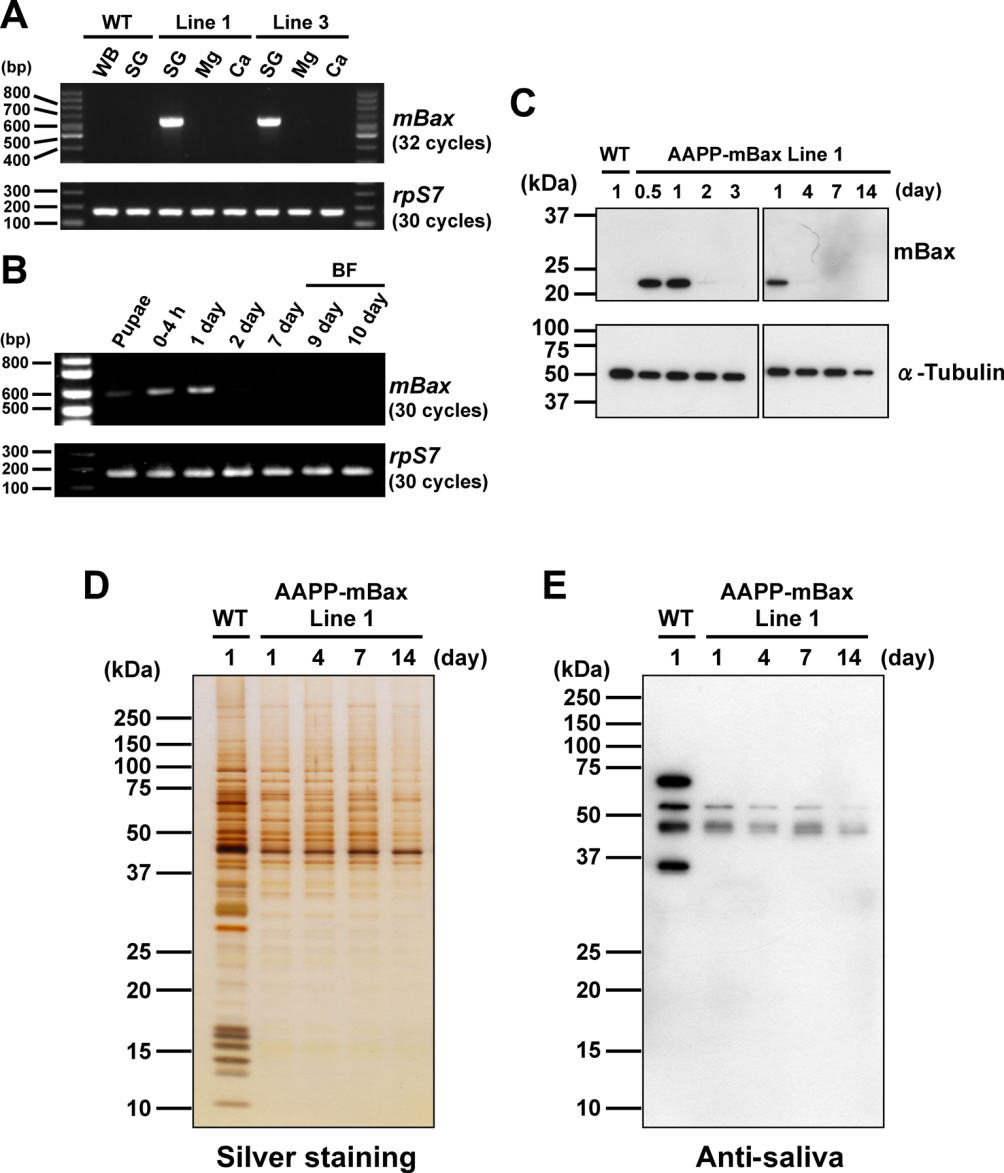
Analysis of transgene expression in salivary glands of female AAPP-mBax lines. (A) Expression of the *T7-mBax* mRNA in the AAPP-mBax mosquito. The salivary glands (SG), midgut (Mg), and carcass (Ca) of 1-day-old adult females were used in analyses. The whole wild-type (WT) female sample (WB) used for analyses was extracted from 1-3-day-old adults. The *ribosomal protein S7* (*rpS7*) gene was used as a control for ubiquitous expression. (B) The expression profile of *T7-mBax* mRNA in AAPP-mBax line 1 female mosquitoes. One-day-old female pupae and female adults (within 4 h, 1 day, 2 days, 7 days, 9 days, 10 days after eclosion) were used in analyses. Nine-day- and 10-day-old adult females were fed blood 7 days after eclosion (BF). Whole female bodies were used in analyses. The *ribosomal protein S7* (*rpS7*) gene was used as a control for ubiquitous expression. (C) Detection of the T7-mBax protein in the salivary gland of AAPP-mBax mosquitoes (line 1) by immunoblotting with anti-T7 antibodies. An anti-alpha-tubulin antibody was used as the loading control. The age of mosquitoes (days post-eclosion) is indicated above. (D) Reductions in the amount of proteins in the salivary glands of female AAPP-mBax mosquitoes (line 1). Silver staining of salivary gland proteins separated by SDS-PAGE. Samples of salivary glands from wild-type (WT) and AAPP-mBax mosquitoes were loaded. The age of mosquitoes (days) is indicated above. (E) The gel loading of the same samples in (D) was analyzed by immunoblotting with the serum of a mouse repeatedly bitten by *An*. *stephensi* (anti-saliva).

**Fig 3:**
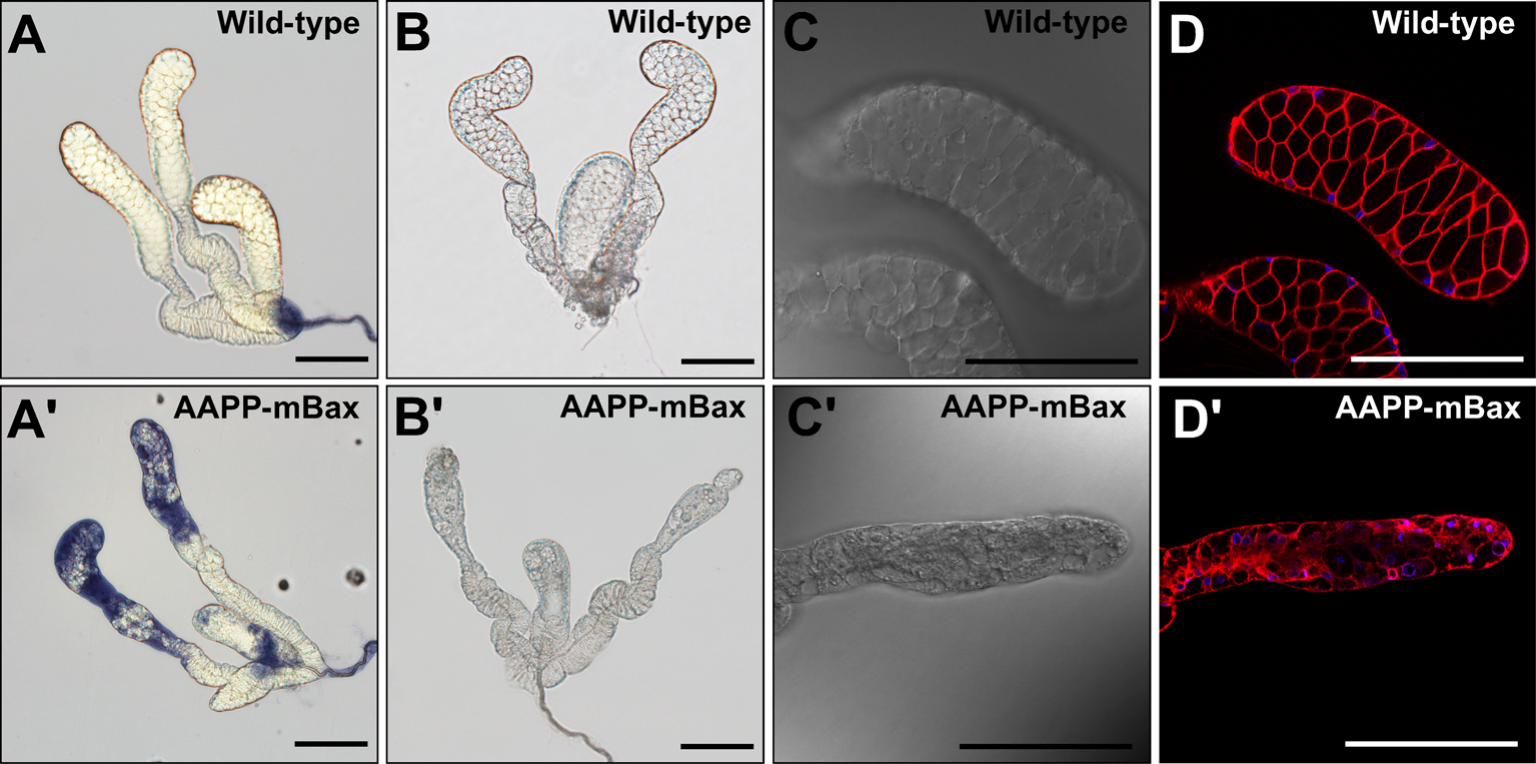
Salivary glands of an AAPP-mBax mosquito. (A, A’) Salivary glands of 3-day-old adult female wild-type mosquitoes and AAPP-mBax mosquitoes stained with trypan blue. (B) A normal salivary gland from a 7-day-old adult female wild-type mosquito. (B’) An abnormal salivary gland with aberrant distal-lateral lobes from a 7-day-old adult female AAPP-mBax mosquito (line 1). (C, C’) Differential interference contrast images of the salivary glands of a 7-day-old adult female wild-type mosquito and AAPP-mBax mosquito (line 1). (D, D’) Fluorescence images of the salivary glands of a 7-day-old adult female wild-type mosquito and AAPP-mBax mosquito stained with CellMask (Red) as a marker for cell membranes and Hoechst 33342 (Blue) as a marker for DNA. Scale bars = 100 μm.

*T7-mBax* mRNA was exclusively expressed in the salivary glands of the two AAPP-mBax lines (Fig 2A), indicating that its expression was adequately controlled by the *aapp* promoter in the two transgenic lines [13]. The expression of *T7-mBax* mRNA was also observed at the pupal stage and immediately after eclosion, but markedly declined 2 days after eclosion (Fig 2B). We then examined T7-mBax expression in the salivary glands of the AAPP-mBax line using an immunoblot analysis with an anti-T7 antibody. Although the T7-mBax protein (Mr = 20 kDa, approximately) was detected in the salivary glands immediately after eclosion, its signal was absent in 3-day-old or older females (Fig 2C). Furthermore, the expression of the *T7-mBax* gene was not induced by blood feeding (Fig 2B), while the expression of other transgenes by the *aapp* promoter exhibited blood feeding-inducible properties [6,13]. The absence of T7-mBax expression in 3-day-old or older females appears to be due to the progression of cell death caused by the expression of mBax. In support of this result, the salivary glands of 3-day-old females in the AAPP-mBax line stained with trypan blue, which is a dye used to stain dead cells, whereas those of wild-type mosquitoes did not (Fig 3A and 3A'). In 7-day-old AAPP-mBax females, the morphology of the distal-lateral lobes was abnormal (Fig 3B and 3B'); however, this abnormality was milder in the medial lobes than in the distal-lateral lobes. Although the distal-lateral lobes of the salivary glands in wild-type mosquitoes shrank immediately after blood feeding due to the discharge of saliva into the host (S2A Fig), the salivary glands of the AAPP-mBax line showed a more abnormal morphology. The abnormal morphology of cells in the distal-lateral lobes was observed in the AAPP-mBax line by staining with CellMask as a marker for cell membranes under confocal microscopy (Fig 3D and 3D’). Cell death was observed in the distal-lateral lobes, which contain large amount of salivary proteins. These results are consistent with our previous findings showing the expression of the non-toxic fluorescent protein DsRed. In these transgenic lines, strong red fluorescence was observed in the distal-lateral lobes [13–15].

We then examined the amount of salivary proteins in these tissues using an SDS-PAGE analysis and immunoblot analysis with anti-saliva antibodies. The amount of salivary proteins increased in wild-type mosquitoes 4 days after eclosion, whereas that in AAPP-mBax line mosquitoes was markedly lower at the same time points (Fig 2D, 2E and S2 Fig). Reductions in protein levels appeared to frequently occur in the low molecular range (less than 37 kDa) (Fig 2D). This reflects the ablation of the distal-lateral lobes. AAPP is one of these smaller proteins and also one of the major salivary components in the distal-lateral lobes. The expression of *aapp* mRNA was significantly reduced in the transgenic lines (S3 Fig). These results suggest that cell death was induced in cells in the distal-lateral lobes, but did not occur in all cells because the production of a small amount of salivary components was detected. Reduction in protein levels in the salivary glands 1 day after eclosion implied that cell death was induced immediately after or before eclosion. This result is supported by the expression of the *mBax* gene already occurring in the pupal stage (Fig 2B). The morphological abnormality in the salivary glands and reductions in salivary protein levels were confirmed in AAPP-mBax line 3 (S4 Fig). Therefore, we used line 1 in subsequent experiments.

### Blood feeding behavior of AAPP-mBax line mosquitoes

**Fig 4:**
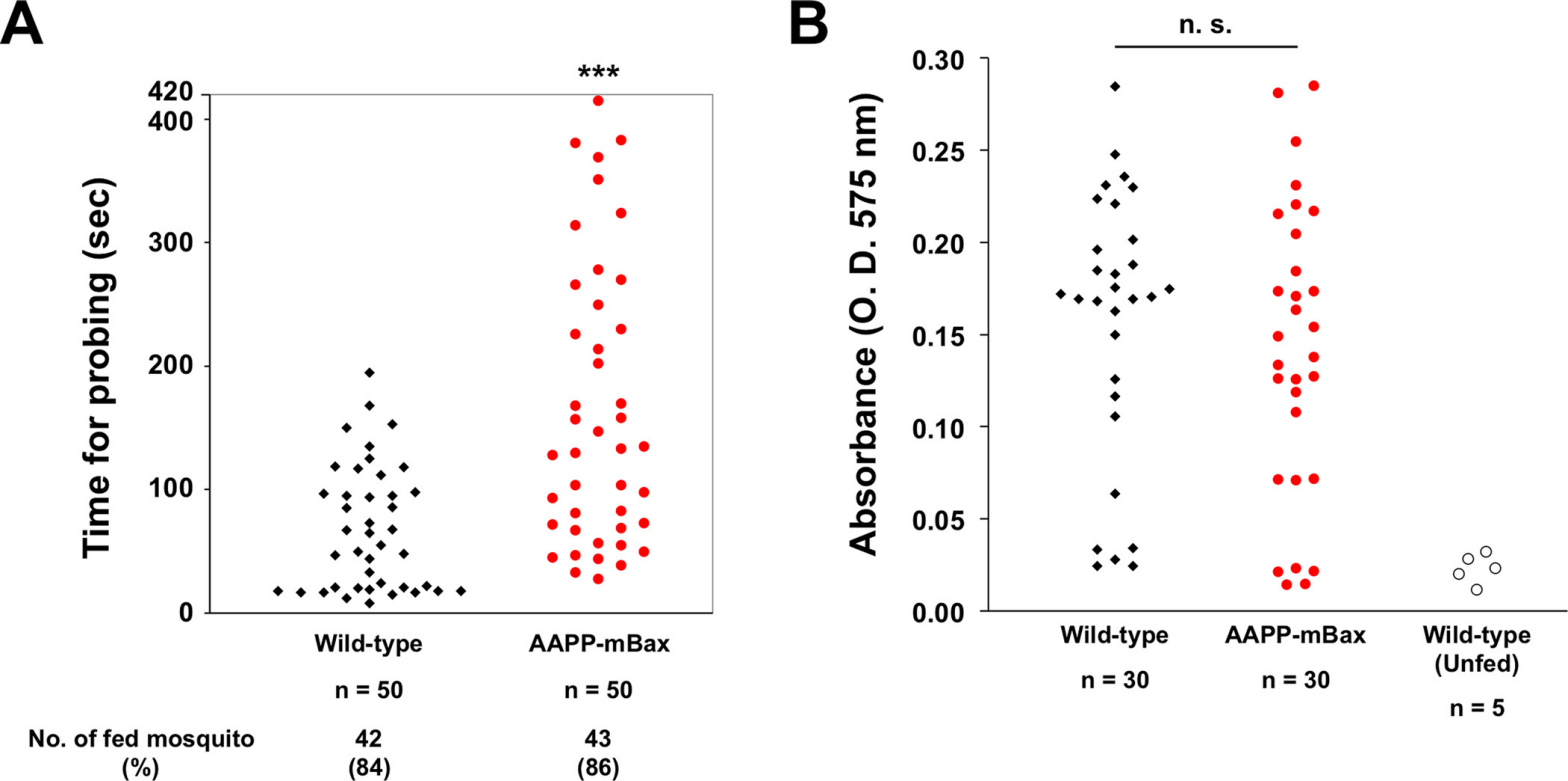
Analysis of the blood feeding behavior of AAPP-mBax line (line 1) mosquitoes. (A) Measurements of probing times by wild-type and AAPP-mBax mosquitoes. Each dot corresponds to one female mosquito. The probing time is defined as the time taken from the initial insertion of the mouthpart into the skin until the initial observation of the ingestion of blood in the abdomen. Probing times were significantly longer in AAPP-mBax mosquitoes than in wild-type mosquitoes. Data from two independent experiments using separate generations of mosquitoes were pooled. The number and ratio of blood-fed mosquitoes within 420 seconds are indicated below (n = 50, ***: *P* < 0.0001, calculated by the Mann-Whitney *U* test). (B) Comparison of the amount of ingested blood between wild-type and AAPP-mBax mosquitoes. The amount of ingested blood was evaluated using hemoglobin contents in the abdomens of mosquitoes allowed access to the same mice for 30 min. The hemoglobin content was represented as OD_575_ units. The abdomens of unfed wild-type mosquitoes were used as the negative control. No significant difference was observed between AAPP-mBax and wild-type mosquitoes. n.s., not significant (*P* = 0.3007, calculated by the Mann-Whitney *U* test).

In an attempt to clarify whether reductions in the amount of saliva in transgenic line mosquitoes affects blood feeding, we examined the probing times of AAPP-mBax and wild-type mosquitoes during blood feeding on mice. Probing times were significantly longer in transgenic mosquitoes than in wild-type mosquitoes (Fig 4A and S5 Fig). However, no significant differences were observed in the number of blood-fed mosquitoes after 7 minutes between AAPP-mBax and wild-type mosquitoes. We also evaluated the amount of ingested blood by measuring the hemoglobin content. No significant differences were noted in hemoglobin contents between transgenic and wild-type mosquitoes following blood feeding on mice for 30 minutes (Fig 4B). These results indicate that reductions in the amount of saliva affect the probing time of mosquitoes; however, AAPP-mBax female mosquitoes still have the ability to ingest blood when there are no time restrictions.

### Transgenic mosquitoes impaired *Plasmodium berghei* development

**Fig 5:**
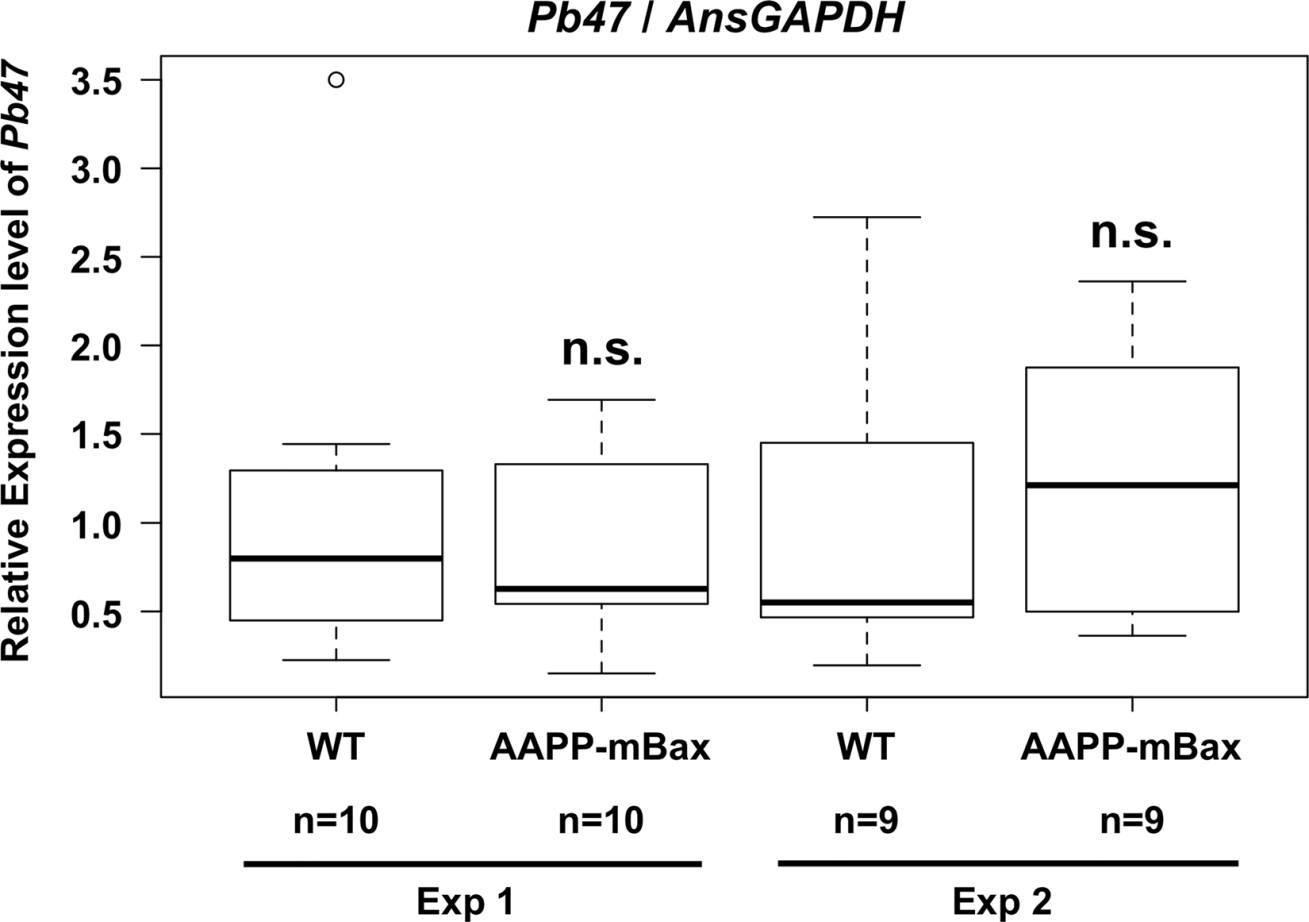
Comparison of the *P*. *berghei* load in the midgut of mosquitoes. The expression of the *P*. *berghei Pb47* gene in wild-type (WT) and AAPP-mBax (line 1) mosquitoes after blood feeding was analyzed by quantitative RT-PCR. Relative expression levels are shown, with the average value of wild-type mosquitoes being 1. The expression levels of *Pb47* were normalized using that of the *An*. *stephensi GAPDH* gene. Two independent experiments were performed. No significant differences were observed between AAPP-mBax and wild-type mosquitoes. n.s., not significant (*P* = 0.9377 in Exp 1 and *P* = 0.9672 in Exp 2).

**Fig 6:**
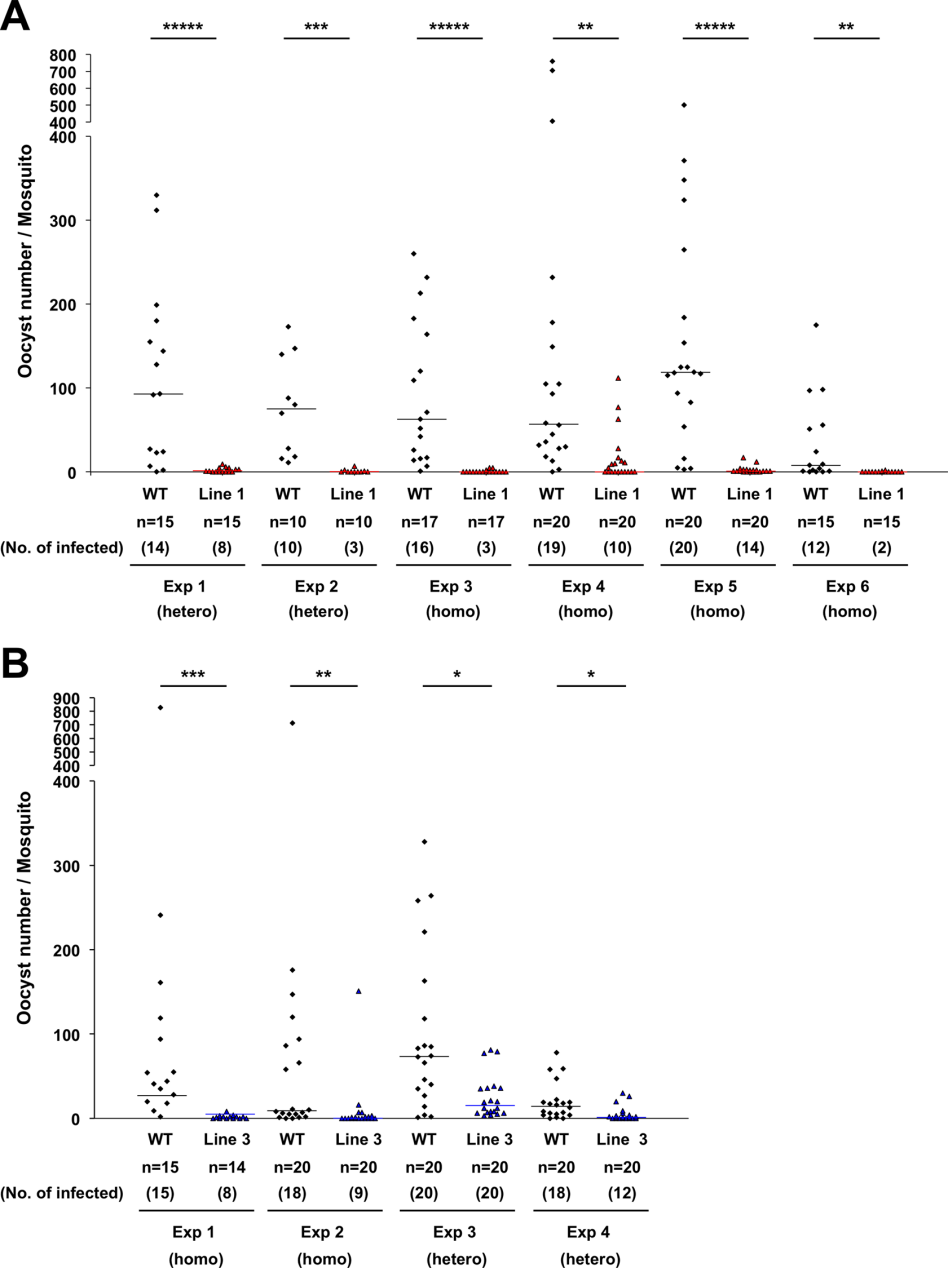
Oocyst numbers of *P*. *berghei* in wild-type and AAPP-mBax mosquitoes. Midguts were dissected 10–12 days after blood feeding and oocyst numbers were counted by microscopy. Homozygous and heterozygous transgenic mosquitoes were tested. (A) Results of line 1. Six independent experiments (Exp 1–6) were shown. (B) Results of line 3. Four independent experiments (Exp 1–4) were shown. The status of transgenic mosquitoes (homozygous or heterozygous) is represented below the numbers of the experiment. These experiments were performed using separate generations of mosquitoes. (*****: *P* < 0.000001, ***: *P* < 0.0001, **: *P* < 0.001, *: *P* < 0.01, calculated by the Mann-Whitney *U* test). The line shows the median.

In order to examine the effects of the abnormal salivary glands of the AAPP-mBax line on malaria infection, we examined malaria parasite development in AAPP-mBax mosquitoes. AAPP-mBax and wild-type female mosquitoes were allowed to feed on the same mice infected with *P*. *berghei* parasites for 30 min. Fully engorged mosquitoes were collected and used in analyses. Under these conditions, no significant differences were observed in the total parasite load or the gametocyte load in the midgut between AAPP-mBax and wild-type mosquitoes in quantitative PCR analyses of *P*. *berghei 18S rRNA* and *P*. *berghei Pb47*, which is a female gametocyte- and gamete-specific gene (Fig 5 and S6 Fig). Despite the intake of the same amount of malaria gametocytes in the midgut, oocyst numbers were significantly lower in AAPP-mBax line 1 mosquitoes than in wild-type mosquitoes (Fig 6A). Similar results were obtained in AAPP-mBax line 3 mosquitoes (Fig 6B). Following reductions in oocyst numbers, the accumulation of sporozoites in the salivary glands was markedly inhibited in transgenic mosquitoes (Table 1). Furthermore, infectious blood-fed AAPP-mBax mosquitoes did not transmit the parasite to mice because of the lack of sporozoites in the salivary glands. (Table 2, S2A and S2B Table). These results strongly suggest that oocyst formation and/or development were inhibited in AAPP-mBax mosquitoes and also that saliva plays an important role in the process leading to oocyst development.

**Table 1:** Inhibition of infection by *P*. *berghei* sporozoites in salivary glands of transgenic mosquitoes.

	No. of mosquitoes^a^													
	Line 1-Exp 1		Line 1-Exp 2		Line 1-Exp 5		Line 1-Exp 6		Line 3-Exp 2		Line 3-Exp 3		Line 3-Exp 4	
No. of sporozoites(spz)/salivary gland		**										***		
	WT	Line 1	WT	Line 1	WT	Line 1	WT	Line 1	WT	Line 3	WT	Line 3	WT	Line 3
0	1	9	1	12	8	25	3	20	2	20	5	18	12	29
1 ≤ spz ≤ 50	0	1^b^	0	0	0	0	0	0	0	0	0	2^c^	1	0
50 < spz ≤ 500	1	0	1	0	6	0	1	0	3	0	6	0	4	0
500 < spz ≤ 5000	4	0	8	0	9	0	7	0	11	0	7	0	9	0
5000 < spz	4	0	5	0	3	0	9	0	4	0	2	0	1	0
No. of infected/total	9/10	1/10	14/15	0/12	18/26	0/25	17/20	0/20	18/20	0/20	15/20	2/20	17/27	0/29
(% of infected mosquitoes)	(90)	(10)	(93)	(0)	(69)	(0)	(85)	(0)	(90)	(0)	(75)	(10)	(63)	(0)

**: *P*<0.001

***: *P*<0.0001 (The Mann-Whitney *U* test).

^a^Mosquitoes in independent experiments (Line 1-Exp 1, 2, 5 and 6 and Line 3-Exp 2–4) were used from the group in the oocyst analysis (Fig 6, Line 1-Exp 1, 2, 5 and 6 and Line 3-Exp 2–4).

^b^Two sporozoites were observed in a pair of salivary glands from a mosquito.

^c^One sporozoite was observed in a pair of salivary glands from a mosquito.

**Table 2:** Blockade of *P*. *berghei* transmission in transgenic mosquitoes.

Vectorial competence^a^ (7 mosquito feedings^b^)	No. of infected/total^c^ (% infected mice)	
	WT	AAPP-mBax Line 1
Exp 1 (day 22)^d^	3/4 (75)	0/3 (0)
Exp 2 (day 20)^d^	5/5 (100)	0/5 (0)
Total	8/9 (88.9)	0/8 (0)

^a^Vectorial competence is an evaluation of a vector’s ability to transmit a parasite to naïve mice. Wild-type (WT) mosquitoes and AAPP-mBax line 1 mosquitoes (homozygous) were allowed to feed on the same *P*. *berghei*-infected mouse for 30 min, and fully engorged mosquitoes were collected. Seven mosquitoes were fed on individual naïve mice on day 22 (Exp 1) or day 20 (Exp 2) after the infectious blood meal for more than 30 min. The infection status of each mouse was evaluated by a microscopic examination of a Giemsa-stained blood smear on days 4, 7, 10, and 17 after mosquito feeding. Mice that had no parasites by day 30 were defined to be uninfected.

^b, c^The infection status of each mouse and mosquito is shown in S2A and S2B Table.

^d^Two independent experiments were performed using separate generations of mosquitoes.

### Exflagellation-inducing activity was reduced in the salivary gland of the AAPP-mBax line

**Fig 7:**
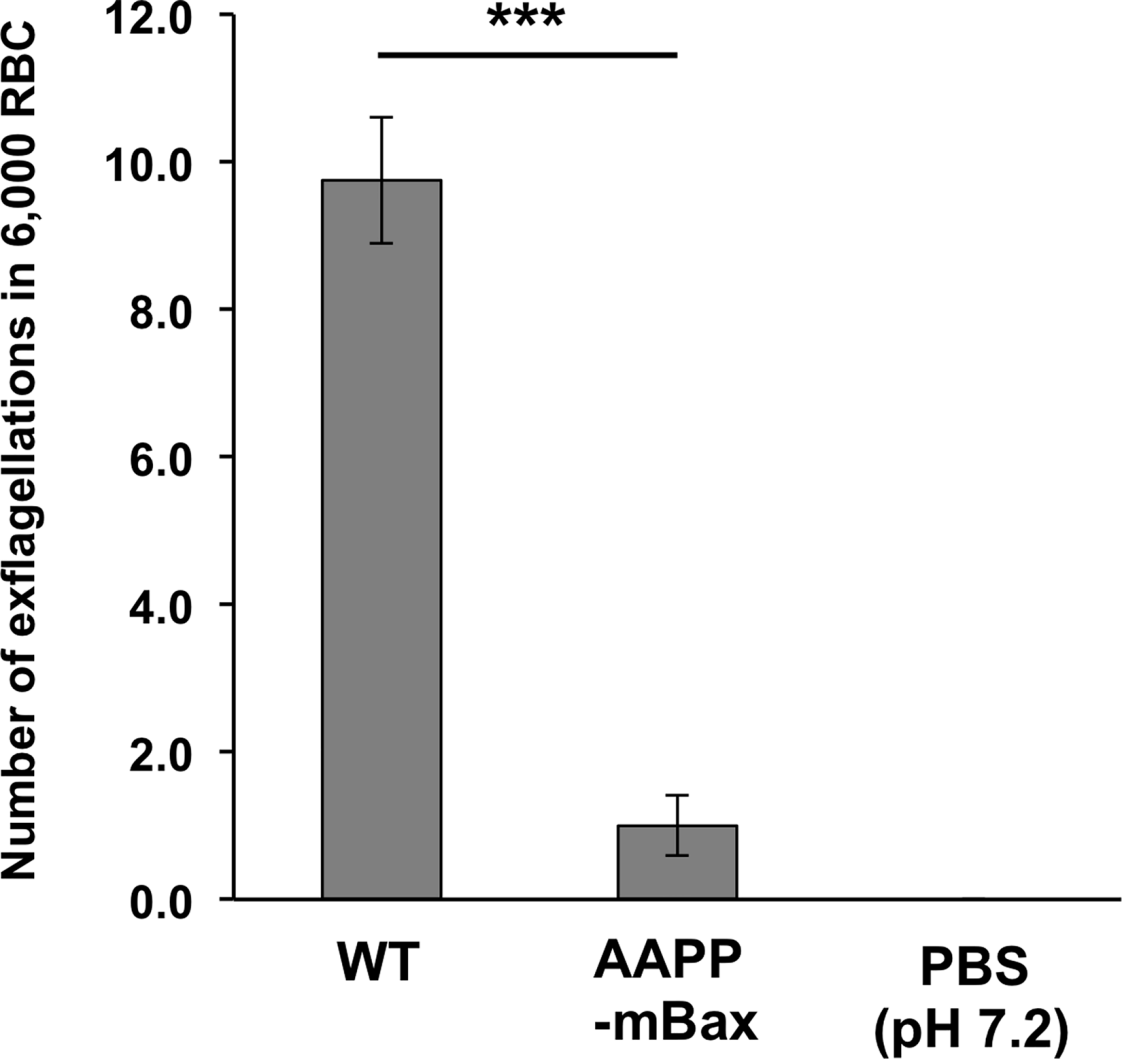
Exflagellation-inducing activity in salivary glands of AAPP-mBax and wild-type mosquitoes. The homogenate of the salivary glands (equivalent to 10 pairs of salivary glands) with PBS (pH 7.2) was mixed with *P*. *berghei*-infected blood. The number of exflagellation bodies per 4 fields (approximately 6,000 RBC) was counted. An experiment using PBS (pH 7.2) was the negative control (n = 4 experiments, ***: *P* < 0.0001, calculated by the Student’s *t*-test).

The salivary glands of anopheline mosquitoes were previously reported to contain a large amount of exflagellation-inducing factors, and the possibility of their uptake via blood feeding and contribution to malaria infectivity in the midgut was demonstrated [26]. We investigated the exflagellation-inducing activity of the salivary glands of AAPP-mBax line mosquitoes against a male gametocyte of *P*. *berghei*. The homogenate of the salivary glands of AAPP-mBax mosquitoes exhibited markedly lower activity in the induction of exflagellation *in vitro* than that of wild-type mosquitoes (Fig 7 and S7 Fig). This result suggests that reductions in exflagellation-inducing factors in saliva inhibit malaria development in the midgut by reducing exflagellation in AAPP-mBax mosquitoes.

## Discussion

In the present study, we produced transgenic *An*. *stephensi* containing distal-lateral lobe-aberrant salivary glands with extremely low amounts of salivary components. We found that oocyst formation in the midgut was markedly inhibited in this transgenic line. The homogenate of the salivary glands of this line suppressed exflagellation, which is an essential event in microgametogenesis before the fertilization of *Plasmodium* in the mosquito midgut. These results indicate that anopheline mosquito saliva plays an important role in blood feeding as well as in interactions with malaria parasites in the midgut. A previous study reported that the inhibition of the surface component of salivary glands, Saglin protein suppressed salivary gland invasion by sporozoites [11]. We herein demonstrated for the first time that the salivary glands are important for the transmission of malaria parasites to the midgut of mosquitoes.

The females of the AAPP-mBax line contained aberrant distal-lateral lobes in their salivary glands due to the expression of the cell death effector mBax under the control of the female-specific-salivary gland promoter, suggesting that cell death is induced in this tissue in transgenic *An*. *stephensi* mosquitoes. Previous studies reported that mBax induced cell death in the *Drosophila* eye [24,25]. In these studies, some of the ommatidia were ablated, but not completely. The phenotypes with the aberrant distal-lateral lobes of the salivary glands in the AAPP-mBax lines were similar to these findings. On the other hand, mBax induced cell death in the *Bombyx* posterior silk gland, and this tissue was completely ablated [18]. This phenotypic variation may be derived from the tissue and/or developmental stage.

The salivary components of anopheline mosquitoes are considered to contain a large number of molecules that facilitate blood feeding, such as those for anti-clotting, anti-platelet aggregation, vasodilation, and anesthesia [7,27]. Our results suggest that saliva plays an important role in the initiation of blood ingestion, in which a mosquito searches for a suitable vessel with its proboscis by penetrating host skin, rather than in the full ingestion of blood. Saliva also plays an important role in probing by *Ae*. *aegypti* [28,29].

We found that oocyst formation in the midgut was markedly inhibited in the AAPP-mBax line. T7-mBax was not expressed in the midgut of AAPP-mBax mosquitoes, and the normal morphology of the midgut was observed using microscopy (Fig 2A and S8 Fig). Therefore, our results indicate that the inhibition of oocyst formation is caused by a reduction in saliva in aberrant salivary glands. A previous study reported exflagellation-inducing activity resulting from gametocyte activation factor (GAF) in mosquitoes [30]. Another study showed that exflagellation-inducing activity was greater in the salivary gland than in the midgut or head of *An*. *stephensi*, indicating that GAF in the salivary glands is injected into the host during blood feeding, is delivered to the midgut together with blood, and then contributes to exflagellation [26]. The results obtained for malaria infectivity in the AAPP-mBax line support this finding. Previous studies proposed the possibility of xanthurenic acid (XA) exhibiting exflagellation-inducing activity in mosquitoes using *in vitro* assays [31,32], whereas other studies suggested that undetermined molecules other than XA play important roles in the induction of exflagellation *in vivo* [33,34]. Multiple molecules may be involved in the induction of exflagellation in mosquitoes. Future studies are needed in order to identify the molecules inducing exflagellation and establish whether these molecules are proteins. A comparative analysis of salivary components between AAPP-mBax line and wild-type mosquitoes, and gene knockout systems using genome-editing tools in mosquitoes [35–38] may lead to the identification of essential molecules for this event, in addition to the subsequent development of new drugs to control vectors and malaria parasites. In the present study, we used the *An*. *stephensi*-*P*. *berghei* system to evaluate the susceptibility of transgenic mosquitoes to malaria infections. The role of saliva in exflagellation using an mBax-mediated cell death system needs to be examined in other mosquito species and human malaria parasite species, such as the *An*. *gambiae*-*P*. *falciparum* system.

Genetic strategies for the population replacement of vector mosquitoes with those that are unable to transmit malaria parasites have recently been proposed as one of the new strategies for malaria control [2,38,39]. Transgenic mosquitoes expressing antimalarial effector molecules, such as a single-chain antibody (scFv) to malaria parasite antigens and antimicrobial peptides, have been reported [6,40–42]. We previously described a transgenic *An*. *stephensi* mosquito expressing 2A10 scFv to the malaria circumsporozoite protein in the salivary gland [6]. This transgenic line inactivated malaria parasites in the salivary glands, and strongly reduced malaria infectivity to mice. However, the transmission of malaria to mice was not completely blocked in this line. In the present study, sporozoite accumulation in the salivary glands of AAPP-mBax mosquitoes was markedly inhibited and did not transmit the parasite to hosts in a rodent malaria model. Therefore, we reconfirmed that the salivary glands is a highly effective target tissue for malaria control strategies. Although no significant difference was observed in the lifespan of mosquitoes between AAPP-mBax line and wild-type mosquitoes (S1 Fig), the probing behavior of female AAPP-mBax mosquitoes was impaired, suggesting that the reproductive fitness of this line may be lower than that of wild vector populations under natural conditions. In the future, genome-editing tools may establish non-transgenic mosquito lines that block the transmission of malaria by disrupting the genes that encode the salivary molecules inducing exflagellation or interactions with sporozoites when these molecules are identified. The use of transgenic mosquitoes expressing transgenes in the salivary gland for vector population replacement strategies has some limitations and must be used with caution because transgene products have the potential to be injected with mosquito saliva into the host skin. This issue does not exist for knockout mosquitoes lacking salivary components.

This cell death technology may be applied to specific deficiencies in a particular tissue and developmental stage depending on the promoter function [18]. Therefore, this system may facilitate the elucidation of vector-parasite interactions in not only the salivary gland, but also the midgut as the site for a fertilization and development of malaria parasites. Moreover, technology inducing functional deficiencies in tissues may be applied to studies on the chemosensory system for host seeking by the olfactory organ, such as the antennae and maxillary palps in adult mosquitoes [43–45]. This system may also lead to the production of sterile mosquitoes for vector control by inducing cell death in gonadal cells. This technology provides a powerful tool for elucidating the functions of mosquito tissues and advancing the development of new vector control strategies.

## Materials and Methods

### Ethics statement

All mouse procedures were approved by the Institutional Animal Experiment Committee of Jichi Medical University (Number: 15101), and in accordance with the Institutional Regulation for Animal Experiments and Fundamental Guidelines for Proper Conduct of Animal Experiment and Related Activities in Academic Research Institutions under the jurisdiction of the Ministry of Education, Culture, Sports, Science and Technology.

### Animals

The *An*. *stephensi* mosquito strain SDA500 was maintained at 26°C and 50–70% relative humidity under 13-h light/11-h dark conditions. Female BALB/c strain mice were obtained from Japan SLC (Hamamatsu, Shizuoka, Japan). The *P*. *berghei* ANKA 234 strain was maintained by cyclical passages through BALB/c mice and *An*. *stephensi* mosquitoes. The tissues of mosquitoes were observed under the inverted microscope, IX73 equipped with a DP73 digital camera (Olympus, Tokyo, Japan).

Regarding fluorescent staining, the dissected salivary glands were immediately stained with 5 μg/l CellMask Plasma Membrane Stain (Life Technologies, Carlsbad, CA, USA) in phosphate-buffered saline (PBS, pH 7.2) for 10 min, and then washed three times. The specimens were mounted in PBS with Hoechst 33342 (NucBlue Live Cell Stain ReadyProbes Reagent, Life Technologies) and observed using the confocal microscope Leica TCS SP5 (Leica, Wetzlar, Germany).

### Plasmid construction

The mouse *Bax* gene (*mBax*) with a T7 tag was amplified by PCR from the *mBax* in the pEF-BOS-T7 vector [18,46] using the primers pAAPP-mBax-startL: 5’-ACAGGTGAATAAACGATGGCCAGCATGACTGGTGG-3’ and TrypolyA-mBax-stopR: 5’- GCCGAGATCGCATGCTCAGCCCATCTTCTTCCAGA-3’. Fragments of the *An*. *stephensi aapp* promoter (pAAPP) and *An*. *gambiae trypsin* terminator (tryter) were amplified by PCR from pMinos-EGFP-aappP-mDsRed-Pbcsp-antryp1T [15] by the primers pAAPP-SalI: 5’-GCGTCGACCACCTTATAAGACGGAGCTC-3’ and pAAPPR-XbaI: 5’-GCTCTAGACGTTTATTCACCTGTGAACTG-3’, TrypolyA-NotI: 5’-GCGGCCGCATGCGATCTCGGCTTCAAAAC-3’ and TrypolyA-SpeI: 5’-ACTAGTCACCCTTCAGCGAAAGCTTGTC-3’, respectively. PCR reactions were performed using KOD plus neo polymerase (Toyobo, Tokyo, Japan). The tryter fragment was digested with *Not* I and *Spe* I, and then cloned into the *Not* I/*Spe* I site of pSLfa1180fa [47] to generate pSL-tryter. The pAAPP fragment was digested with *Sal* I and *Xba* I, and then cloned into the *Sal* I/*Xba* I site of pSL-tryter to generate pSL-pAAPP-tryter. The pAAPP-tryter fragment was excised from pSL-pAAPP-tryter by digestion with *Asc* I and *Fse* I, and was then cloned into the *Asc* I/*Fse* I site of pBac[3xP3-EGFPaf] [47] to generate pBac[pAAPP-tryter; 3xP3-EGFP]. The *T7-mBax* fragment was cloned between pAAPP and tryter of pBac[pAAPP-tryter; 3xP3-EGFP] using the GENEART seamless cloning and assembly kit (Life Technologies, Carlsbad, CA, USA) to generate the pBac[pAAPP-mBax; 3xP3-EGFP] transformation vector. Procedures for the microinjection of vectors into embryos, screening of transgenic individuals, and generation of homozygous lines have been described previously [48]. We used homozygous transgenic mosquitoes in experiments.

### Southern blotting

The isolation of genomic DNA from mosquitoes and the Southern blotting analysis were performed as described previously [49]. Genomic DNA was digested with *Msp* I, separated on a 0.8% agarose gel, and then transferred to a Hybond-N+ membrane (GE Healthcare UK Ltd., Buckinghamshire, UK). Probe labeling and the detection of signals were performed using AlkPhos Direct labeling Reagents and CDP-Star Detection Reagent (GE Healthcare UK Ltd.) according to the supplier’s protocol.

### Inverse polymerase chain reaction (inverse PCR)

Inverse PCR was performed as described previously [18]. Genomic DNA was extracted from adult females using a DNeasy Blood and Tissue kit (Qiagen, Hinden, Germany). DNA was digested with *Msp*I, and then circulated with T4 DNA ligase (New England Biolabs, Ipswich, Ma, USA) at 4°C overnight. In order to amplify the left-hand region, the primers for the first PCR were ks129: 5'-AAATCAGTGACACTTACCGCATT-3' and ks133: 5'-ACTATAACGACCGCGTGAGTCAA-3', followed by ks130: 5'-CGACTGAGATGTCCTAAATGCAC-3' and ks395: 5'-TTATCGATACCGTCGACCTCGAC-3' for the second PCR.

### SDS-PAGE and immunoblotting

Groups of 10 pairs of salivary glands were homogenized by a plastic homogenizer in 100 μl of sample buffer (Nacalai Tesque, Kyoto, Japan) containing 5% 2-mercaptoethanol, and were then boiled at 95°C for 3 min. Ten microliters of each sample (equivalent to 1 pair of salivary glands) was separated on a 12% NuPAGE gel (Life Technologies). Silver staining of the gel was performed using the Sil-best stain one kit (Nacalai Tesque).

Regarding immunoblotting, a rabbit anti-T7 tag monoclonal antibody (T7 tag, D9E1X XP rabbit mAb, #13246, Cell Signaling, MA, USA) and rabbit anti-alpha-tubulin monoclonal antibody (11H10 mAb, #2125, Cell Signaling) were used as primary antibodies. The sera of mice repeatedly bitten by *An*. *stephensi* were used as anti-saliva antibodies for the detection of saliva. Mice were routinely used to feed wild-type *An*. *stephensi*. Feeding by mosquitoes was repeated more than 10 times and mice were subjected to a total of approximately 1,500 bites. Each sample (equivalent to 1 pair of salivary glands) was separated on a 12% NuPAGE gel, and then transferred to a Hybond ECL Membrane (GE Healthcare UK Ltd.). Membranes were blocked with T-TBS (20 mM Tris-HCl, 137 mM NaCl, pH 7.4, 0.05% Tween-20) containing 5% skimmed milk (Megmilk Snow Brand, Tokyo, Japan). Membranes were incubated with primary antibodies. The polypeptides recognized by primary antibodies were detected with either horseradish peroxidase (HRP)-conjugated goat anti-rabbit IgG (#7074, Cell Signaling) or HRP-conjugated goat anti-mouse IgG (Life Technologies). The detection of HRP-labeled antibodies was performed by exposing membranes on Hyperfilm-ECL using ECL Prime Western Blotting Detection Reagents (all GE Healthcare UK Ltd.) according to the supplier’s protocol.

### Reverse transcription-polymerase chain reaction (RT-PCR)

Total RNA was extracted using TRIzol and purified using the PureLink RNA mini kit (all Life Technologies). Two hundred and fifty nanograms of total RNA was reverse-transcribed from each sample using the High-Capacity cDNA Reverse Transcription kit (Life Technologies). cDNA was used for the PCR amplification of the *mBax* and *An*. *stephensi ribosomal protein S7* (*rpS7*) genes using Taq DNA polymerase (New England Biolabs). The primers pAAPP-mBax-startL: 5’-ACAGGTGAATAAACGATGGCCAGCATGACTGGTGG-3’ and TrypolyA-mBax-stopR: 5’-GCCGAGATCGCATGCTCAGCCCATCTTCTTCCAGA-3’ were used to amplify *mBax*. The primers AnsS7L: 5’-GGCGATCATCATCTACGTGC-3’ and AnsS7R: 5’-CGGTCTCTTCTGCTTGTTGG-3’ were used to amplify *rpS7*.

Regarding quantitative RT-PCR, cDNA was used for the PCR amplification of *An*. *stephensi aapp* and *An*. *stephensi glyceraldehyde 3-phosphate dehydrogenase (GAPDH)* genes using LightCycler-FastStart DNA Master SYBR Green I plus (Roche Diagnostics GmbH, Mannheim, Germany). Reactions were analyzed with a LightCycler instrument (Roche). The primers AAPP-QPCR-F: 5’-AAACCGGTGCCGATGCTGGT-3’ and AAPP-QPCR-R: 5’-ACCTTCCTCGCCTGCCTCGT-3’ were used to amplify *aapp*. The primers GAPDH-Aste-QPCR-F: 5’-GCCGTCGGCAAGGTCATCCC-3’ and GAPDH-Aste-QPCR-R: 5’-TTCATCGGTCCGTTGGCGGC-3’ were used to amplify *GAPDH*. Relative expression levels of the *aapp* gene were normalized using expression levels of the *GAPDH* gene. Data were analyzed using the Student’s *t*-test.

### Probing time analysis

The probing times of mosquitoes were measured based on previously described methods [50]. Mosquitoes were starved of sugar the night before the tests, and individually caged in polystyrene tubes (70 x 120 mm) with a hole (20 mm in diameter) and cotton net. Mice were anesthetized with pentobarbital (50 mg/kg, intraperitoneally), and a part of the shaved ventral skin of these mice made contact with the hole in the cage containing mosquitoes. The probing time was defined as the time taken from the initial insertion of mouthparts into the skin until the initial observation of the ingestion of blood in the abdomen. The observation of probing times was terminated after 420 sec, and the times recorded were used in analyses. Data were analyzed using the Mann-Whitney *U* test.

### Analysis of the amount of blood ingested

The amounts of hemoglobin in the abdomens of blood-fed mosquitoes were measured with a Hemoglobin colorimetric assay kit (Cayman, Ann Arbor, MI, USA). The abdomens of mosquitoes after blood feeding were dissected and homogenized in 200 μl of a hemoglobin detector solution. Samples were incubated at 25°C for 15 min, and then centrifuged for 10 min at 20,000 x *g*. Absorbance at 575 nm of 100 μl of cleared samples was measured in a 96-well microtiter plate using SpectraMax M5 (Molecular Devices, Sunnyvale, CA, USA). Data were analyzed using the Mann-Whitney *U* test.

### Quantification of the parasite load in the midgut

Transgenic and wild-type mosquitoes 10–14 days after eclosion were allowed to feed on the same *P*. *berghei*-infected mouse for 30 min. Only fully engorged mosquitoes were collected and used for DNA extraction within 1 hour. Genomic DNA was extracted using a DNeasy Blood and Tissue kit (Qiagen). Genomic DNA was used for the PCR amplification of the *P*. *berghei 18S ribosomal RNA* (*Pb18S*) gene and *An*. *stephensi GAPDH* gene using LightCycler-FastStart DNA Master SYBR Green I plus (Roche). Reactions were analyzed with a LightCycler instrument (Roche). The primers Pb18S-F: 5’-GGAGATTGGTTTTGACGTTTATGTG-3’ and Pb18S-R: 5’-AAGCATTAAATAAAGCGAATACATCCTTAC-3’ were used to amplify *Pb18S*. The primers GAPDH-Aste-QPCR-F and GAPDH-Aste-QPCR-R were used to amplify *GAPDH*. Relative expression levels of the *Pb18S* gene were normalized using *GAPDH* gene expression levels. Regarding quantitative RT-PCR for *Pb47*, the extraction of total RNA, synthesis of cDNA, and PCR amplification were performed using the methods described above. The primers Pb47F-QPCR: 5’- ATTGCCTTGGTATGCCCCAA-3’ and Pb47R-QPCR: 5’- TGTGTGCGCTGTCTTCAGAT-3’ were used to amplify *Pb47*. The primers GAPDH-Aste-QPCR-F and GAPDH-Aste-QPCR-R were used to amplify *GAPDH*. Relative expression levels of the *Pb47* gene were normalized using expression levels of the *GAPDH* gene. Data were analyzed using the Student’s *t*-test.

### Malaria infection assay

Transgenic and wild-type mosquitoes 10–14 days after eclosion were allowed to feed on the same *P*. *berghei*-infected mouse for 30 min. Only fully engorged mosquitoes were collected and fed 5% fructose at 21°C. On days 10–12, midguts were dissected, and the number of oocysts per midgut was then counted under a phase contrast microscope. On days 19–21, salivary glands were dissected, and the number of sporozoites per salivary gland was then measured using previously described methods [42,51]. In assays using homozygous transgenic mosquitoes, transgenic and wild-type mosquitoes came from two separately raised populations. In assays using heterozygous transgenic mosquitoes, transgenic and wild-type mosquitoes were taken from a single heterogeneous strain using EGFP selection. Data were analyzed using the Mann-Whitney *U* test.

In order to transmit *P*. *berghei* to mice, 7 mosquitoes were fed on individual naïve mice on day 22 (Exp 1) or day 20 (Exp 2) after the infectious blood meal for more than 30 min. The infection status of each mouse was evaluated by a microscopic examination of a Giemsa-stained tail vein blood smear. Mice that had no parasites by day 30 were defined to be uninfected.

### Exflagellation induction assay

The exflagellation induction assay was performed as described previously [26]. Groups of 20 pairs of salivary glands were homogenized in 20 μl of PBS (pH 7.2), and centrifuged at 20,000 x *g* for 10 min. The supernatant (1 pair of salivary glands /μl) was used in the assay. Tail blood was collected from a *P*. *berghei*-infected mouse with gross parasitemia of 30–55%. Ten microliters of the supernatant (equivalent to 10 pairs of salivary glands) was mixed with 0.5 μl of infected blood. One microliter of the mixture was immediately mounted on a glass slide with highly water-repellent rimmed 5-mm diameter holes (Matsunami, Osaka, Japan) under a cover slip, and incubated at 21°C for 15 min. Exflagellation bodies were counted using x400 magnification under a microscope in a total of 4 fields [approximately 6,000 erythrocyte (RBC: red blood cells)]. Data were analyzed using the Student’s *t*-test or Mann-Whitney *U* test.

### Accession numbers for genes

The following genes were analyzed in this study: *mouse Bax* (*mBax*, GenBank NM_007527.3), *ribosomal protein S7* (*rpS7*, GenBank AF539918.2), *anopheline antiplatelet protein* (*aapp*, GenBank AB212871.1), *glyceraldehyde-3-phosphate dehydrogenase* (*GAPDH*, GenBank AB753163.1), *Plasmodium berghei 18S rRNA* (*Pb18S*, GenBank M19712.1), and *Pb47* (GenBank AF314253.1).

## Supporting Information

S1 Fig Comparison of survival rates between AAPP-mBax (line 1) and wild-type mosquitoes. (A) Analysis under the sugar feeding only condition. The same number of mosquitoes (n = 30) immediately after eclosion was used in analyses. The survival curves of the groups were estimated by Kaplan-Meier methods. No significant difference was observed in females or males between AAPP-mBax and wild-type mosquitoes (Female; *P* = 0.3823 and Male; *P* = 0.7142, calculated by the Log-rank test). (B) Analysis under the condition of blood feeding twice in females. The black arrows represent when a blood meal was given. No significant difference was observed in females between AAPP-mBax and wild-type mosquitoes (*P* = 0.5898, calculated by the Log-rank test).
(TIF)

S2 Fig Analysis of salivary glands of female wild-type mosquitoes. (A) Salivary glands of blood-fed wild-type mosquitoes. Salivary glands dissected from unfed female wild-type mosquitoes (WT) and blood-fed female wild-type mosquitoes (post-blood feeding: PBF) (7-day-old mosquitoes) were shown. The salivary glands of blood-fed female mosquitoes were dissected within 1 h of blood feeding. Scale bars = 100 μm. (B) Silver staining of salivary gland proteins separated by SDS-PAGE. Samples from wild-type (WT) mosquitoes were loaded. The age of mosquitoes (days) is indicated above. (C) The gel was analyzed by immunoblotting with anti-*An*. *stephensi* saliva antibodies (anti-saliva).
(TIF)

S3 Fig Relative expression levels of the *aapp* gene in 1-day-old adult female salivary glands in wild-type (WT) and AAPP-mBax (lines 1 and 3) mosquitoes by a quantitative RT-PCR analysis. Relative expression levels are shown and the value of wild-type mosquitoes was 1. The expression levels of *aapp* were normalized using the expression levels of *GAPDH*. (n = 3 experiments, ***: *P* < 0.0001, calculated by the Student’s *t*-test).
(TIF)

S4 Fig Analysis of salivary glands of AAPP-mBax line 3 females. (A) Abnormal salivary gland with aberrant distal-lateral lobes in a 7-day-old adult female. Scale bar = 100 μm. (B) Detection of the mBax protein in the salivary glands of females by immunoblotting with anti-T7 and anti-alpha-tubulin antibodies. Homogenate samples of the salivary glands were used in analyses. An anti-alpha-tubulin antibody was used as the loading control. The age of mosquitoes (days post eclosion) is indicated above. (C) Reductions in the amount of proteins in the salivary glands of females. Silver staining of salivary gland proteins separated by SDS-PAGE. Samples of the salivary glands from wild-type (WT) and AAPP-mBax (line 3) mosquitoes were loaded. The age of mosquitoes (days) is indicated above.
(TIF)

S5 Fig Measurements of probing times by wild-type and AAPP-mBax mosquitoes. Experiments were performed using separate generations of mosquitoes from the sample in Fig 4. Each dot corresponds to one female mosquito. The number and ratio of blood-fed mosquitoes within 420 seconds are indicated below (n = 46, ***: *P* < 0.0001, calculated by the Mann-Whitney *U* test).
(TIF)

S6 Fig Comparison of the *P*. *berghei* load in midguts of wild-type (WT) and AAPP-mBax (line 1) mosquitoes after blood feeding by a quantitative PCR analysis. Relative abundances are shown, with the average value of wild-type mosquitoes being 1. The abundance of the *P*. *berghei 18S rRNA* (*Pb18S*) gene was normalized using the abundance of *An*. *stephensi GAPDH*. Two biological replicates were shown. No significant differences were observed between AAPP-mBax and wild-type mosquitoes (n = 10 mosquitoes, Exp 1; *P* = 0.7482 and Exp 2; *P* = 0.8762 calculated by the Student’s *t*-test).
(TIF)

S7 Fig Exflagellation-inducing activities of salivary glands of AAPP-mBax and wild-type mosquitoes. The homogenate of the salivary glands with PBS (pH 7.2) was mixed with *P*. *berghei*-infected blood. The homogenate of the salivary glands and blood from mice with other parasitemia differed from the sample in Fig 7. The number of exflagellation bodies per 4 fields (approximately 6,000 RBC) was counted. An experiment using PBS (pH 7.2) was the negative control. (n = 4 experiments, *P* < 0.05, calculated by the Mann-Whitney *U* test).
(TIF)

S8 Fig Midguts of the AAPP-mBax mosquito. (A, B) Midguts of 7-day-old adult female wild-type and AAPP-mBax mosquitoes. (C, D) Midguts of 7-day-old adult female wild-type and AAPP-mBax mosquitoes were stained with trypan blue.
(TIF)

S1 Table Comparison of the number of eggs laid between wild-type and AAPP-mBax mosquitoes. (PDF)

S2 Table The infection status of individual mice fed on by wild-type and AAPP-mBax mosquitoes. (PDF)
