# Supplementary figures and images for: Inhibition of Malaria Infection in Transgenic Anopheline Mosquitoes Lacking Salivary Gland Cells
Source: PLoS Pathog. 2016 Sep 6;12(9):e1005872. doi: 10.1371/journal.ppat.1005872 (PMC5012584; doi:10.1371/journal.ppat.1005872)

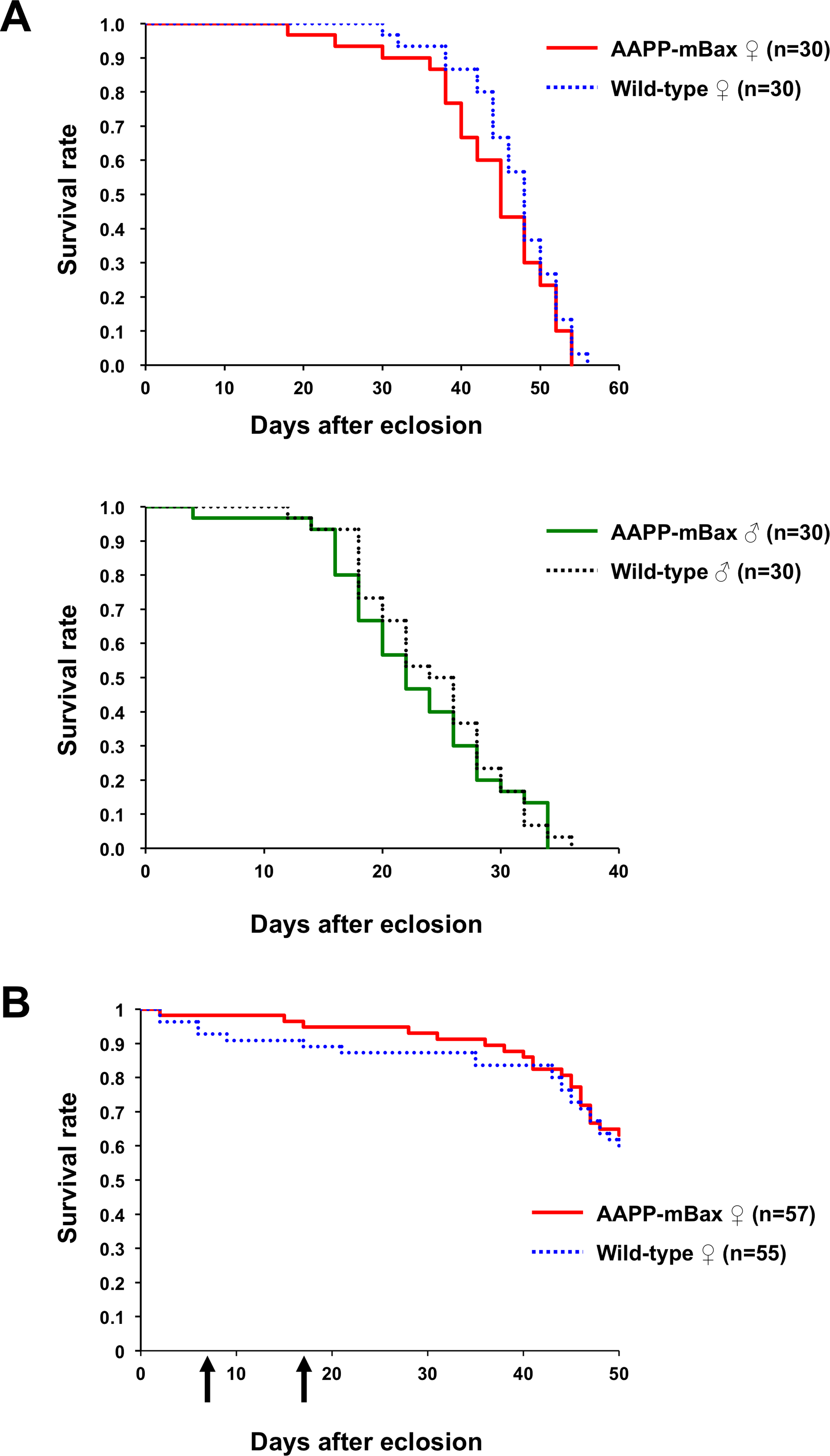

Supplement: S1 Fig — (A) Analysis under the sugar feeding only condition. The same number of mosquitoes (n = 30) immediately after eclosion was used in analyses. The survival curves of the groups were estimated by Kaplan-Meier methods. No significant difference was observed in females or males between AAPP-mBax and wild-type mosquitoes (Female; P = 0.3823 and Male; P = 0.7142, calculated by the Log-rank test). (B) Analysis under the condition of blood feeding twice in females. The black arrows represent when a blood meal was given. No significant difference was observed in females between AAPP-mBax and wild-type mosquitoes (P = 0.5898, calculated by the Log-rank test). (TIF) [file ppat.1005872.s001.tif]

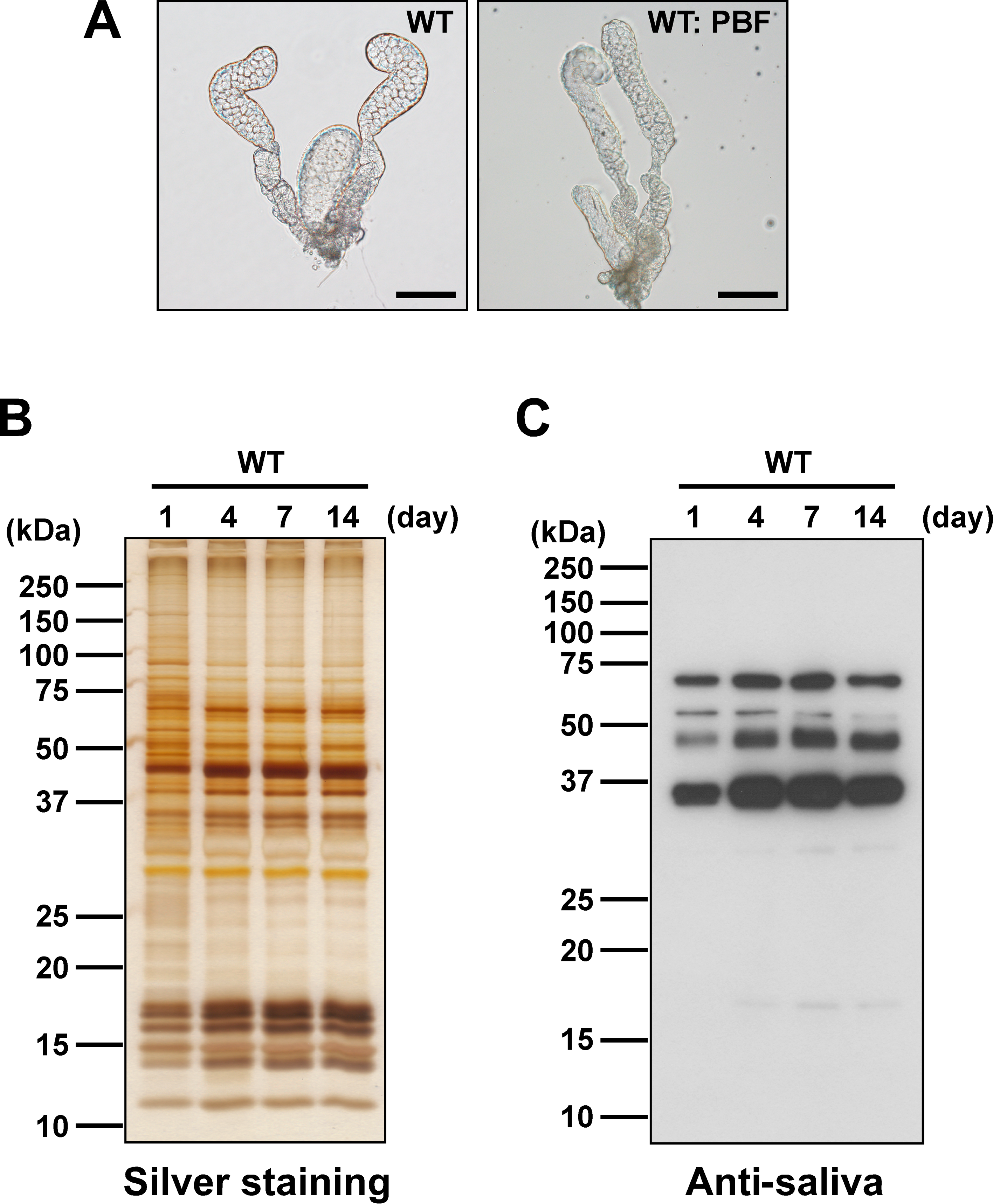

Supplement: S2 Fig — (A) Salivary glands of blood-fed wild-type mosquitoes. Salivary glands dissected from unfed female wild-type mosquitoes (WT) and blood-fed female wild-type mosquitoes (post-blood feeding: PBF) (7-day-old mosquitoes) were shown. The salivary glands of blood-fed female mosquitoes were dissected within 1 h of blood feeding. Scale bars = 100 μm. (B) Silver staining of salivary gland proteins separated by SDS-PAGE. Samples from wild-type (WT) mosquitoes were loaded. The age of mosquitoes (days) is indicated above. (C) The gel was analyzed by immunoblotting with anti-An. stephensi saliva antibodies (anti-saliva). (TIF) [file ppat.1005872.s002.tif]

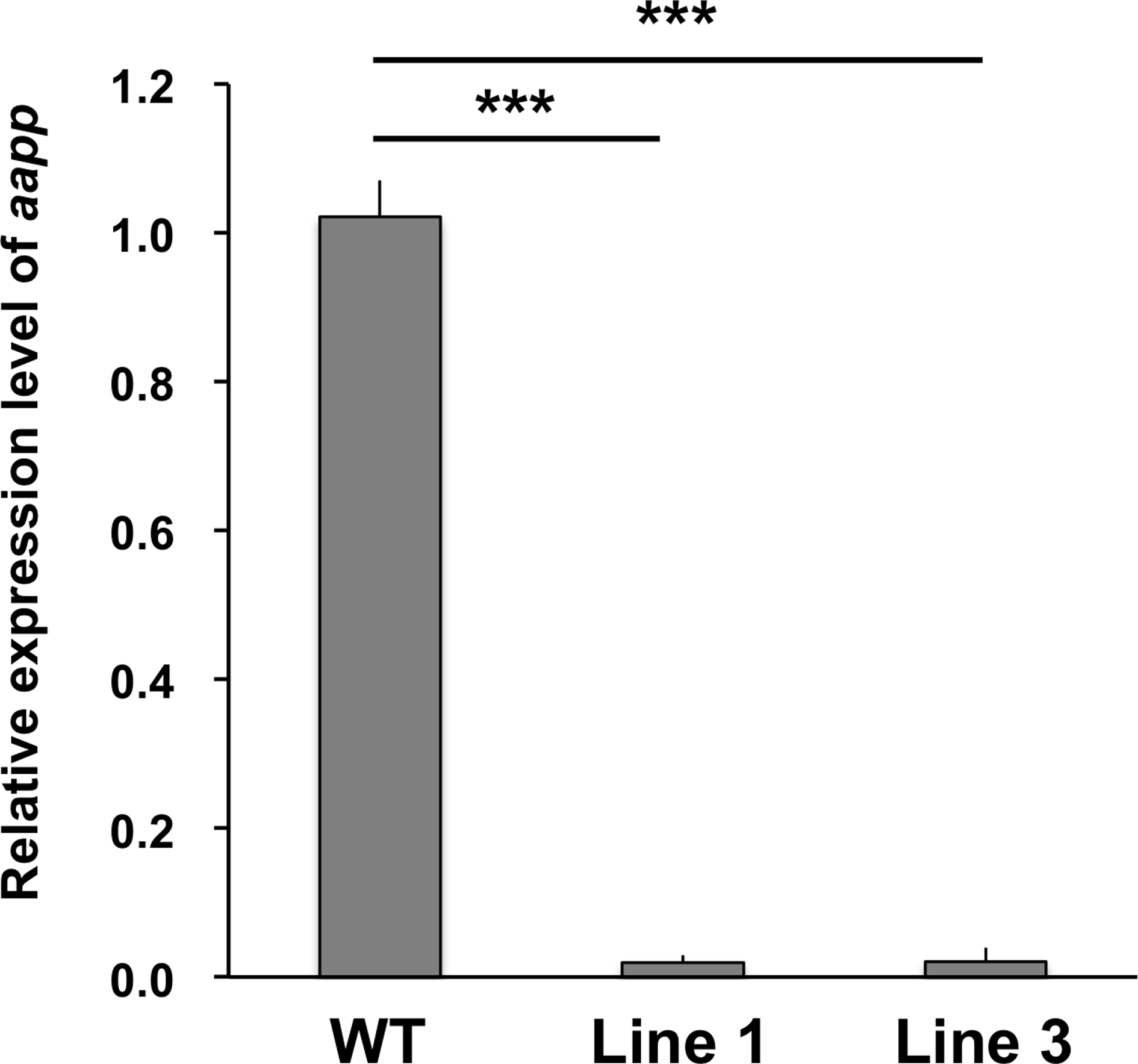

Supplement: S3 Fig — Relative expression levels are shown and the value of wild-type mosquitoes was 1. The expression levels of aapp were normalized using the expression levels of GAPDH. (n = 3 experiments, ***: P < 0.0001, calculated by the Student’s t-test). (TIF) [file ppat.1005872.s003.tif]

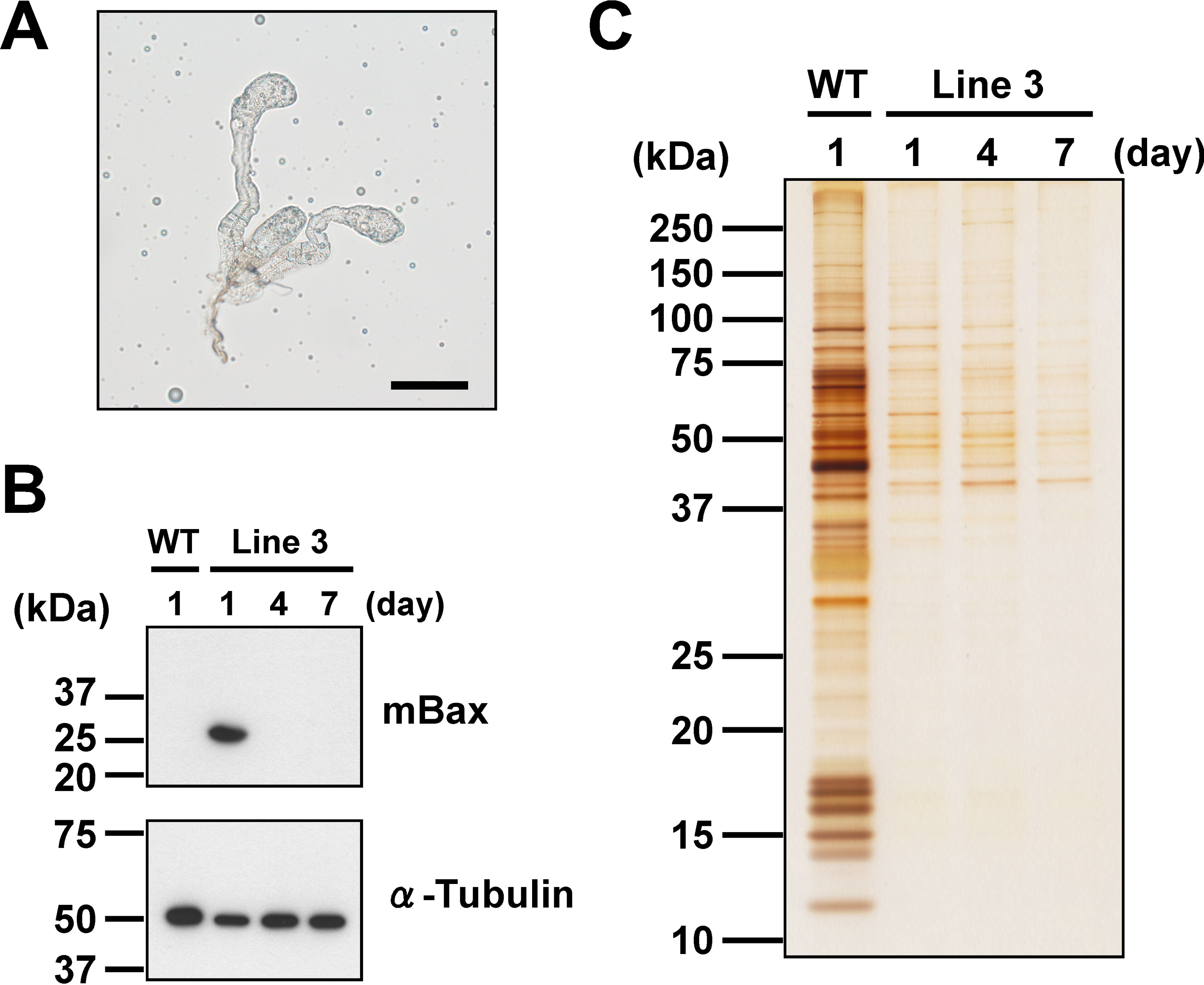

Supplement: S4 Fig — (A) Abnormal salivary gland with aberrant distal-lateral lobes in a 7-day-old adult female. Scale bar = 100 μm. (B) Detection of the mBax protein in the salivary glands of females by immunoblotting with anti-T7 and anti-alpha-tubulin antibodies. Homogenate samples of the salivary glands were used in analyses. An anti-alpha-tubulin antibody was used as the loading control. The age of mosquitoes (days post eclosion) is indicated above. (C) Reductions in the amount of proteins in the salivary glands of females. Silver staining of salivary gland proteins separated by SDS-PAGE. Samples of the salivary glands from wild-type (WT) and AAPP-mBax (line 3) mosquitoes were loaded. The age of mosquitoes (days) is indicated above. (TIF) [file ppat.1005872.s004.tif]

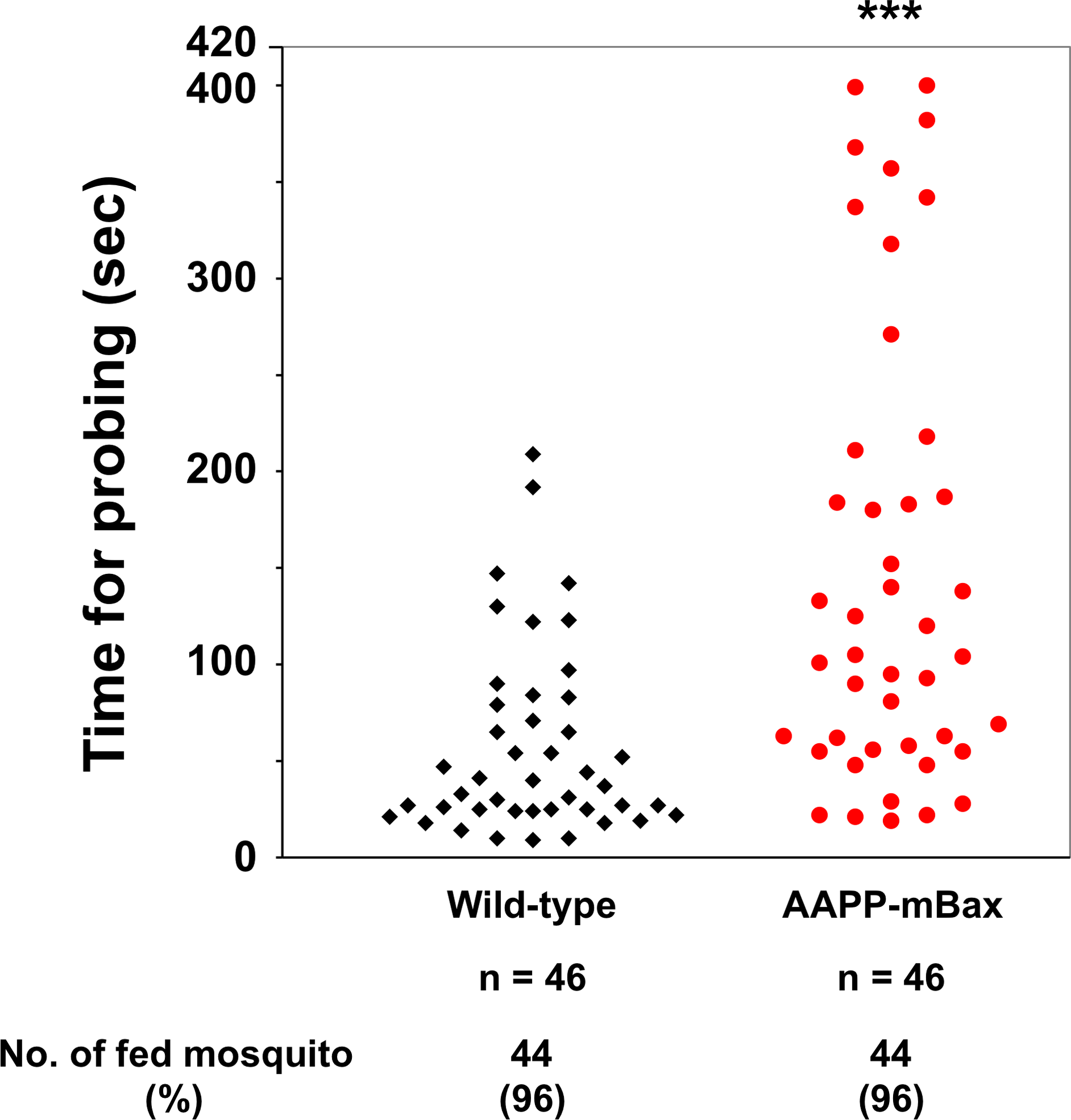

Supplement: S5 Fig — Experiments were performed using separate generations of mosquitoes from the sample in Fig 4. Each dot corresponds to one female mosquito. The number and ratio of blood-fed mosquitoes within 420 seconds are indicated below (n = 46, ***: P < 0.0001, calculated by the Mann-Whitney U test). (TIF) [file ppat.1005872.s005.tif]

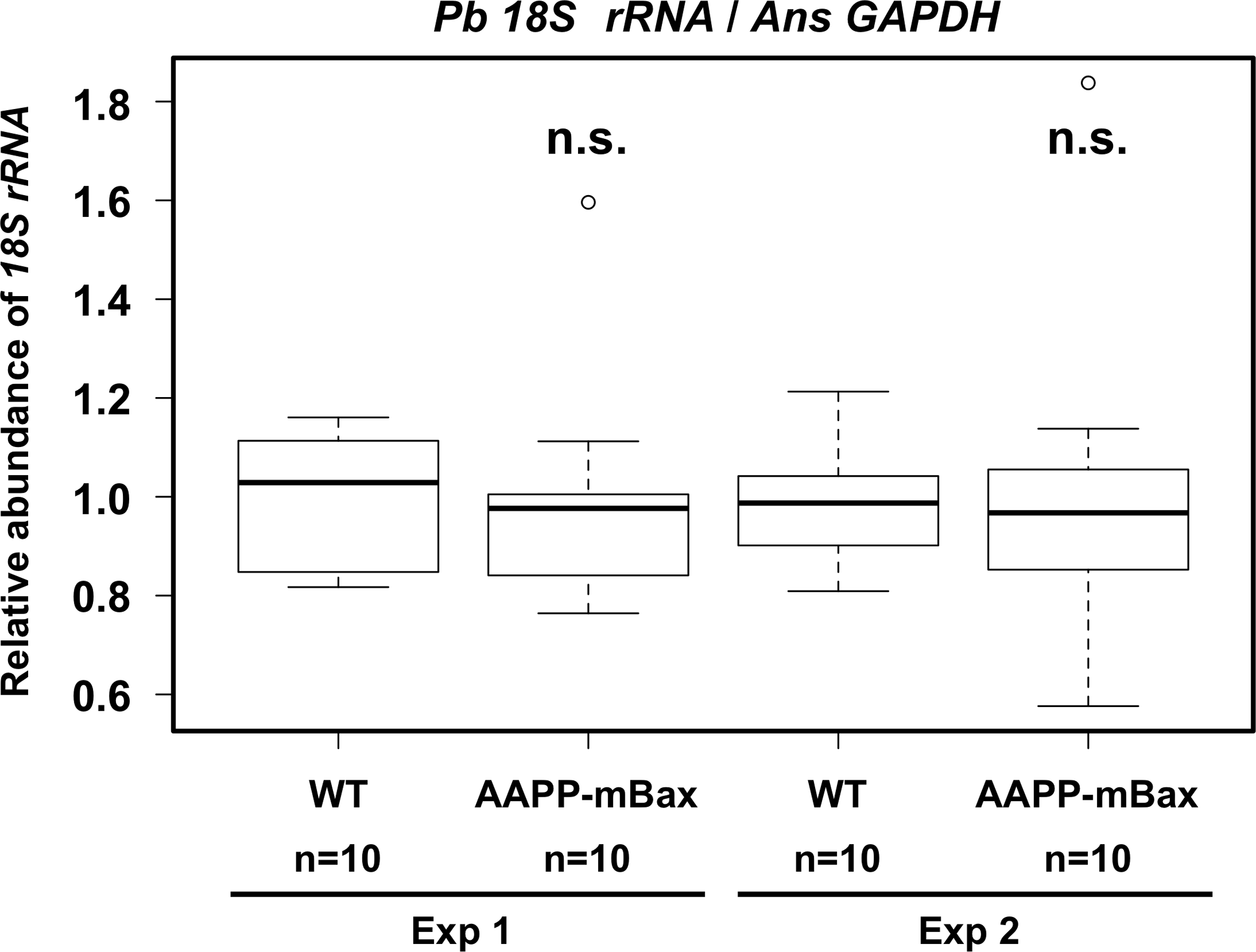

Supplement: S6 Fig — Relative abundances are shown, with the average value of wild-type mosquitoes being 1. The abundance of the P. berghei 18S rRNA (Pb18S) gene was normalized using the abundance of An. stephensi GAPDH. Two biological replicates were shown. No significant differences were observed between AAPP-mBax and wild-type mosquitoes (n = 10 mosquitoes, Exp 1; P = 0.7482 and Exp 2; P = 0.8762 calculated by the Student’s t-test). (TIF) [file ppat.1005872.s006.tif]

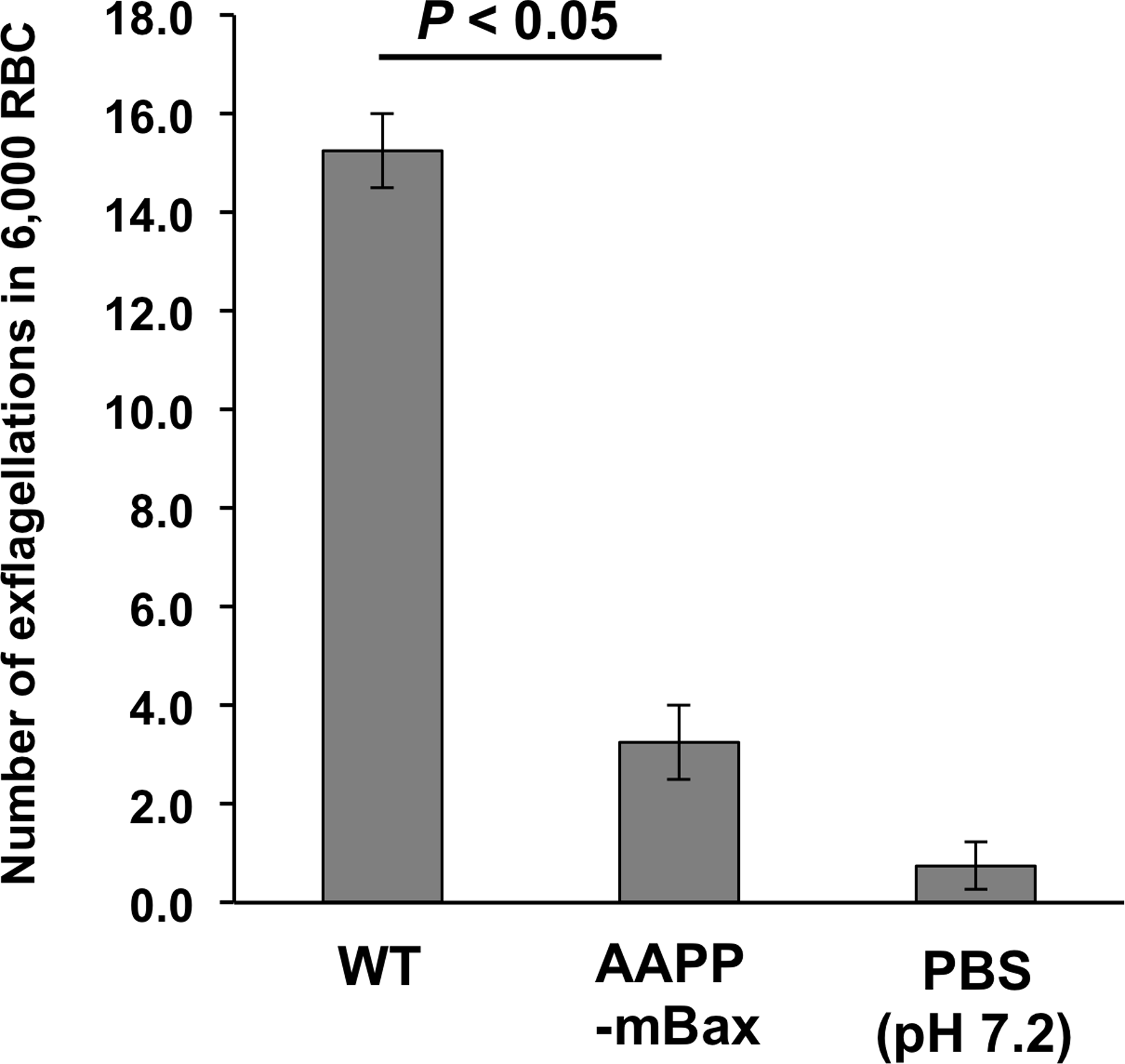

Supplement: S7 Fig — The homogenate of the salivary glands with PBS (pH 7.2) was mixed with P. berghei-infected blood. The homogenate of the salivary glands and blood from mice with other parasitemia differed from the sample in Fig 7. The number of exflagellation bodies per 4 fields (approximately 6,000 RBC) was counted. An experiment using PBS (pH 7.2) was the negative control. (n = 4 experiments, P < 0.05, calculated by the Mann-Whitney U test). (TIF) [file ppat.1005872.s007.tif]

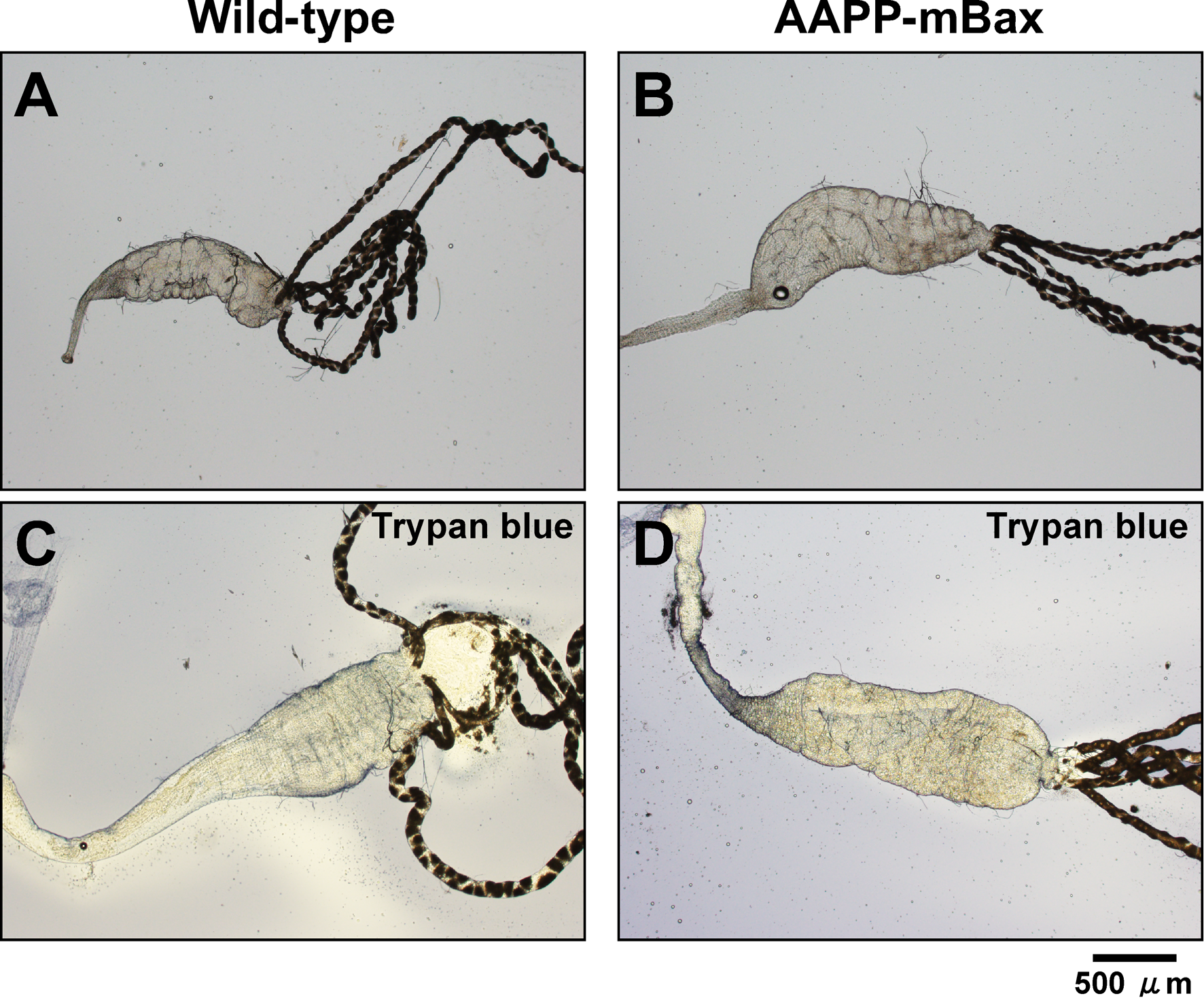

Supplement: S8 Fig — (A, B) Midguts of 7-day-old adult female wild-type and AAPP-mBax mosquitoes. (C, D) Midguts of 7-day-old adult female wild-type and AAPP-mBax mosquitoes were stained with trypan blue. (TIF) [file ppat.1005872.s008.tif]
