# Supplementary material for: Inhibition of Malaria Infection in Transgenic Anopheline Mosquitoes Lacking Salivary Gland Cells
Source: PLoS Pathog. 2016 Sep 6;12(9):e1005872. doi: 10.1371/journal.ppat.1005872 (PMC5012584; doi:10.1371/journal.ppat.1005872)
Supplement: S1 Table — (PDF) [file ppat.1005872.s009.pdf]

S1 Table. Comparison of the number of eggs laid between wild-type and AAPP-mBax mosquitoes.

Group	Wild-Type (n = 19)	AAPP-mBax (n = 18)
Number of eggs*, ** (mean ± SEM)	90.5 ± 7.2	89.8 ± 9.2
Number of females laid hatched eggs	17	16
Hatchability[§], [¶] (mean ± SEM)	0.57 ± 0.08	0.69 ± 0.09
Range of egg hatchability	0 – 0.98	0 – 1.00

*: Four- to seven-day-old female adults were fed the blood of mice, and females were allowed to lay eggs individually 4 to 7 days after blood feeding.

**: There was no significant difference between transgenic and wild-type mosquitoes. $P = 0.9489$ (the Student's *t*-test).

[§]: Average fraction of hatched eggs

[¶]: There was no significant difference between transgenic and wild-type mosquitoes. $P = 0.2606$ (the Mann-Whitney *U* test).
