# Supplementary material for: Inhibition of Malaria Infection in Transgenic Anopheline Mosquitoes Lacking Salivary Gland Cells
Source: PLoS Pathog. 2016 Sep 6;12(9):e1005872. doi: 10.1371/journal.ppat.1005872 (PMC5012584; doi:10.1371/journal.ppat.1005872)
Supplement: S2 Table — (PDF) [file ppat.1005872.s010.pdf]

S2A Table. The infection status of individual mice fed on by wild-type mosquitoes.

| Experiment No. | Mouse I.D. | Infection status of the mouse[a] | Infection status of 7 blood-fed wild-type mosquitoes | | | | | |
|---|---|---|---|---|---|---|---|---|
| | | | Number of sporozoites (spz)/salivary gland[b] | | | | | |
| | | | 0 | 1 ≤ spz ≤ 50 | 50 < spz ≤ 500 | 500 < spz ≤ 5000 | 5000 < spz | unknown[c] |
| EXP 1 | 1-A | Uninfected | 6 | | | | 1 | |
| | 1-B | Infected | 1 | | 2 | 3 | 1 | |
| | 1-C | Infected | 3 | | | | 3 | 1 |
| | 1-D | Infected | 2 | | | 4 | 1 | |
| EXP 2 | 2-A | Infected | | | 1 | 5 | 1 | |
| | 2-B | Infected | 2 | | 2 | 2 | 1 | |
| | 2-C | Infected | 1 | | 2 | 2 | 2 | |
| | 2-D | Infected | 1 | | 1 | 4 | 1 | |
| | 2-E | Infected | | | 1 | 3 | 3 | |
| Total | | 8/9 | 16 | | 9 | 23 | 14 | 1 |

[a]Seven mosquitoes were allowed to feed on individual naïve mice for more than 30 min. The infection status of each mouse was established by a microscopic examination of a Giemsa-stained blood smear. Mice that had no parasites by day 30 were defined to be uninfected.

[b]Following blood feeding, the salivary glands of mosquitoes were dissected and the number of sporozoites was counted.

[c]It was not possible to count the number of sporozoites because of missing salivary glands.

S2B Table. The infection status of individual mice fed on by AAPP-mBax mosquitoes.

| Experiment No. | Mouse I.D. | Infection status of the mouse[a] | Infection status of 7 blood-fed AAPP-mBax mosquitoes | | | | |
|---|---|---|---|---|---|---|---|
| | | | Number of sporozoites (spz)/salivary gland[b] | | | | |
| | | | 0 | 1 ≤ spz ≤ 50 | 50 < spz ≤ 500 | 500 < spz ≤ 5000 | 5000 < spz |
| EXP 1 | 1-E | Uninfected | 7 | | | | |
| | 1-F | Uninfected | 7 | | | | |
| | 1-G | Uninfected | 7 | | | | |
| EXP 2 | 2-F | Uninfected | 7 | | | | |
| | 2-G | Uninfected | 7 | | | | |
| | 2-H | Uninfected | 7 | | | | |
| | 2-I | Uninfected | 7 | | | | |
| | 2-J | Uninfected | 7 | | | | |
| Total | | 0/8 | 56 | | | | |

[a]Seven mosquitoes were allowed to feed on individual naïve mice for more than 30 min. The infection status of each mouse was established by a microscopic examination of a Giemsa-stained blood smear. Mice that had no parasites by day 30 were defined to be uninfected.

[b]Following blood feeding, the salivary glands of mosquitoes were dissected and the number of sporozoites was counted.
